# Stable Soil Microbial Functional Structure Responding to Biodiversity Loss Based on Metagenomic Evidences

**DOI:** 10.3389/fmicb.2021.716764

**Published:** 2021-10-07

**Authors:** Huaihai Chen, Kayan Ma, Yu Huang, Zhiyuan Yao, Chengjin Chu

**Affiliations:** ^1^State Key Laboratory of Biocontrol, School of Ecology, Sun Yat-sen University, Shenzhen, China; ^2^School of Civil and Environmental Engineering, Ningbo University, Ningbo, China; ^3^State Key Laboratory of Biocontrol, School of Life Sciences and School of Ecology, Sun Yat-sen University, Guangzhou, China

**Keywords:** biodiversity loss, ecosystem function, metabolism, nutrient-cycling, functional stability

## Abstract

Anthropogenic disturbances and global climate change are causing large-scale biodiversity loss and threatening ecosystem functions. However, due to the lack of knowledge on microbial species loss, our understanding on how functional profiles of soil microbes respond to diversity decline is still limited. Here, we evaluated the biotic homogenization of global soil metagenomic data to examine whether microbial functional structure is resilient to significant diversity reduction. Our results showed that although biodiversity loss caused a decrease in taxonomic species by 72%, the changes in the relative abundance of diverse functional categories were limited. The stability of functional structures associated with microbial species richness decline in terrestrial systems suggests a decoupling of taxonomy and function. The changes in functional profile with biodiversity loss were function-specific, with broad-scale metabolism functions decreasing and typical nutrient-cycling functions increasing. Our results imply high levels of microbial physiological versatility in the face of significant biodiversity decline, which, however, does not necessarily mean that a loss in total functional abundance, such as microbial activity, can be overlooked in the background of unprecedented species extinction.

## Introduction

Species loss caused by human activities exceeds natural background levels by several orders of magnitude (Pimm et al., 1995; Purvis et al., 2000). It is therefore essential to understand the consequences of biodiversity decline in ecosystem processes and functioning (Purvis and Hector, 2000; Dıaz et al., 2003). As a result of global biodiversity loss, heterogeneous species are replaced by homogenous thrivers (McKinney and Lockwood, 1999), leading to biotic homogenization at global scales (Olden et al., 2004). Most studies simulating species loss use the random and trait-independent extinction models (Naeem et al., 1994; Tilman et al., 1996, 1997a,1997b; Van Der Heijden et al., 1998; Hector et al., 1999; Kennedy et al., 2002) and assume that species can go extinct in any order. However, biodiversity decline is generally nonrandom (Purvis et al., 2000; Solan et al., 2004), because the few “winners” that replace many “losers” are not randomly distributed in taxonomy or ecological groups (McKinney and Lockwood, 1999; Olden et al., 2004). Thus, studies that adopted a directed species loss model, such as experiments that nonrandomly remove species or functional types from established communities (Dıaz et al., 2003; Chen et al., 2020), are more powerful for discerning how extinction realistically affect ecosystem functioning.

Despite being the major regulator for global biogeochemical cycles, the contribution of microbial diversity to ecosystem functions has been obscured until the last decade (Philippot et al., 2013; Wagg et al., 2014; Bastida et al., 2016; Delgado-Baquerizo et al., 2016b). Understanding the relationship between microbial composition and function at a global scale is essential for predicting changes in ecosystem function under various environmental disturbances (Torsvik and Ovreas, 2002; Wellington et al., 2003; McGill et al., 2006). Although microbial communities are highly heterogeneous, their overall functions have been found to be similar (Louca et al., 2016), possibly attributable to the functional redundancy of soil microbes (Rosenfeld, 2002; Allison and Martiny, 2008). The extent of decoupling between taxonomy and function may also differ between “general” ecosystem processes carried out by a wide range of microbes, such as substrate decomposition (Yin et al., 2000; Rousk et al., 2009; Banerjee et al., 2016), and “special” functions specialized by particular microorganisms (Schimel and Körner, 1995; Balser et al., 2002), such as methane production. Yet, most microbial species loss studies used a random extinction model to create a gradient of microbial diversity achieved by serial dilutions (Peter et al., 2011; Philippot et al., 2013; Delgado-Baquerizo et al., 2016a). Hence, our knowledge on how soil biodiversity loss and simplification of soil community composition influence microbial functional profiles across the globe is still limited.

With the advances in molecular biological technologies, metagenomics have been increasingly used as a promising tool (Tringe et al., 2005) for studying the relationship between functional and taxonomic diversities (Fierer et al., 2012a,b, 2013; Pan et al., 2014; Leff et al., 2015; Souza et al., 2015). Using metagenomics, the abundance of each gene can be assigned to a particular process, and numerous ecosystem functions can be examined simultaneously in one soil sample (Allison and Martiny, 2008). The assessment of multiple functions at the same time acknowledge the importance of multifunctionality (Hector and Bagchi, 2007) and can avoid overestimating functional redundancy (Gamfeldt et al., 2008). To date, open-source web servers are publicly available for metagenomic analyses of taxonomic and functional diversities at global scales (Nelson et al., 2016; Ramírez-Flandes et al., 2019), which enable *in silico* evaluation of changes in functional profiles responding to microbial species loss. Thus, a synthetic metagenome-enabled estimate of microbial community and function resulting from biotic homogenization is urgently needed.

**FIGURE 1:**
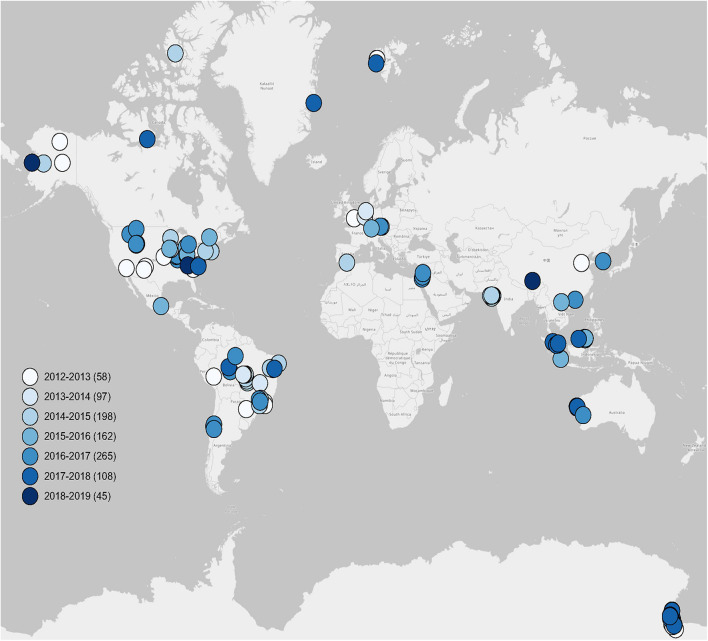
Global distribution of soil metagenomes. Locations of 933 soil metagenomes from 56 publications used in this study. Legends show seven groups of publication periods. Sample sizes of each group are given in parentheses.

**FIGURE 2:**
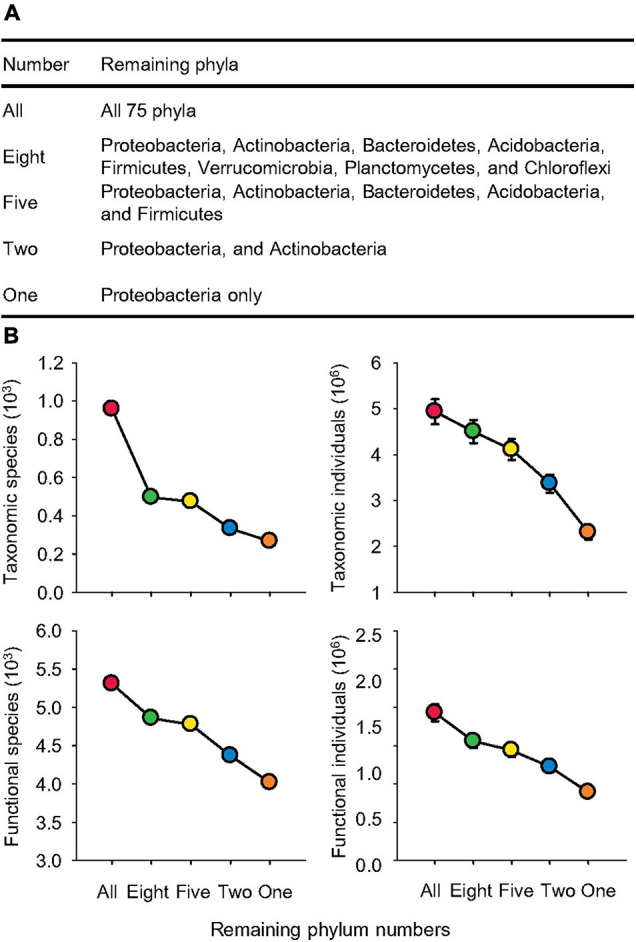
Significant species loss. **(A)** Names of remaining phyla following sequential species loss. **(B)** The species (S) and individuals (N) of taxonomic compositions at the genus levels and functional categories at the function levels affected by sequential species loss.

Here, we constructed five pairs of taxonomic and functional datasets to evaluate five levels of sequential species loss based on 933 soil metagenomes publicly available from 56 MG-RAST studies published in 56 peer-reviewed papers (Figures 1, 2A and Supplementary Table 1). On the basis of this global metagenomic study, we tested our hypotheses that: (1) compared to dramatic taxonomic variation, microbial functional structures are resilient to biodiversity loss, and (2) microbial homogenization caused differential responses in functional profiles between “general” and “special” processes.

## Materials and Methods

### Data Collection

To ensure the quality and integrity of the collected metagenomic data, we only used soil metagenomes that have been published in peer-reviewed publications to construct our metagenomic dataset. Using the key words, such as “soil metagenome,” “shotgun sequencing,” and “MG-RAST,” we obtained a total of 933 soil metagenomes from the MG-RAST server based on 56 peer-reviewed publications from 2012 to 2019 around the world (Figure 1), which have directly submitted their soil shotgun sequences with/without assembly to the MG-RAST database with public accessibility. Detailed information is given in Supplementary Table 1, including Study ID, MG-RAST ID, sample name, publication, latitude (LAT) and longitude (LONG), base pair, sequence hits of taxonomy, and function. The geographic coordinates of LAT and LONG of each soil metagenome were directly originated from publications or metadata in the MG-RAST server.

We downloaded data of soil taxonomic compositions and functional categories at each level based on the information of Study ID and/or MG-RAST ID from the MG-RAST server. We used the RefSeq (Tatusova et al., 2014; phylum, class, order, family, and genus levels) database as taxonomic compositions and the Subsystems (Overbeek et al., 2013; 1, 2, 3, and function levels) database as functional profiles. We preferred the Subsystems database over the KEGG Orthology (Kanehisa et al., 2015), Clusters of Orthologous Groups (Galperin et al., 2015), and Non-supervised Orthologous Groups (Huerta-Cepas et al., 2016) databases, because it has diverse classifications at level 1, allowing comparison of significant variation among different functional categories at this level.

We performed analyses using default settings in the MG-RAST server. Specifically, we set the maximum *e*-value cutoff at 1e-5, minimum identity cutoff at 60%, and minimum alignment length at 50 (Meyer et al., 2008). To construct a sequential reduction of microbial species, we added each major phylum to the database step by step in descending order of their relative abundance. Specifically, to generate a functional profile with only one phylum of Proteobacteria, in the “Analysis” of the MG-RAST server, we chose “Subsystems” as “source,” selected “function” as “level,” and added “Proteobacteria,” the taxonomic phylum that we are interested, in “taxonomy filter” with “RefSeq” as source, and “phylum” as level. In the same way, Proteobacteria and Actinobacteria were selected to represent two remaining phyla. Five remaining phyla were Proteobacteria, Actinobacteria, Bacteroidetes, Acidobacteria, and Firmicutes. Eight remaining phyla were Proteobacteria, Actinobacteria, Bacteroidetes, Acidobacteria, Firmicutes, Verrucomicrobia, Planctomycetes, and Chloroflexi. Thus, we created functional profiles of five levels of sequential species loss named by the remaining phylum numbers, which were all 75 phyla (All), 8 phyla (Eight), 5 phyla (Five), 2 phyla (Two), and 1 phylum (One; Figure 2A).

### Statistical Analyses

Our soil metagenomic data were collected from studies conducted in different locations with different sampling sizes and sequencing depths, which may lead to some bias to our results. To overcome such limitation and minimize bias in sequencing depths among different studies, we standardized the data to relative abundance by dividing hits of taxonomic compositions and functional categories at each level by total hits. Based on the relative abundance of functional categories at the function level and taxonomic compositions at the genus level, we further calculated Bray–Curtis similarity to construct a pairwise Bray–Curtis similarity matrix in PRIMER 7 (Plymouth Routines in Multivariate Ecological Research Statistical Software, v7.0.13, PRIMER-E Ltd., Plymouth, United Kingdom; Clarke and Gorley, 2015). Based on the pairwise Bray–Curtis similarity matrix, we conducted principal coordinates analysis (PCoA) and one-factor permutational multivariate analysis of variance (PERMANOVA) of the main test (pseudo-F statistics) and pair-wise test (pseudo-t statistics) in PRIMER 7 to examine how beta-diversity of the taxonomic composition at the genus level (Taxonomy) and functional profiles at the function level (Function) was affected by sequential species loss. To show the normalized relative abundance of all functional categories at level 1 affected by sequential removal of phyla, we constructed a heat map using dendrograms of hierarchical cluster analysis HeatMapper (Babicki et al., 2016) to group functions based on “Average Linkage” as the clustering method and “Spearman Rank Correlation” as the distance measurement method. To assess the effects of species loss on the beta-diversity of different functions, we separated functional profiles by functional categories at level 1. Based on the relative abundance of functional categories at the function level in each function, we further calculated Bray–Curtis similarity and performed one-factor PERMANOVA of the main test in PRIMER 7 to estimate the statistical significance (pseudo-F) in the beta-diversity of different functions affected by species loss. We used Pearson’s correlations to assess the relationships between the reduction of species and individuals in taxonomy and function, as well as the statistical significance (pseudo-F) of the changes in different functions and their relative abundance along sequential species loss.

To examine the potential interactions of taxonomic compositions and functional categories across the globe, we performed the co-occurrence network analysis using the Molecular Ecological Network Analyses Pipeline^1^ (Zhou et al., 2011; Deng et al., 2012). We standardized the relative abundance of taxonomic compositions at the genus level and functional categories at level 3 to meet the pipeline’s requirements and further submitted it to construct the network with default settings, including: (1) only keeping the species present in more than a half of all samples; (2) only filling with 0.01 in blanks with paired valid values; (3) taking the logarithm with the recommended similarity matrix of Pearson’s correlation coefficient; and (4) calculation order to decrease the cutoff from the top using regress Poisson distribution only. We generated a default cutoff value (similarity threshold, *S*_*t*_) for the similarity matrix to assign a link between the pair of species. Then, we ran the global network properties, the individual nodes’ centrality, and the module separation and modularity calculations based on default settings using greedy modularity optimization. We exported and visualized network files using the Cytoscape software (Shannon et al., 2003).

## Results

### Significant Reduction of Both Microbial Taxonomic and Functional Species

Reducing microbial phylum numbers from all 75 to only 8 dominant bacterial phyla significantly decreased the taxonomic species by 48% (Figure 2B). Further removing three phyla (Verrucomicrobia, Planctomycetes, and Chloroflexi) only triggered a reduction in taxonomic species by 5%. However, these two steps of species loss each caused a decrease in taxonomic individuals, which is the total number of hits of all species, by 9%. Further removals of three phyla (Bacteroidetes, Acidobacteria, and Firmicutes) diminished the taxonomic species by 30% and individuals by 18%. Reduction from two phyla (Proteobacteria and Actinobacteria) to only Proteobacteria led to the greatest reduction in taxonomic individuals by 32% and a decline in species by 20%. Thus, the decrease in taxonomic individuals (Pearson’s correlation *r*^2^ = 0.95, *p* < 0.0001) was more linear than species (Pearson’s correlation *r*^2^ = 0.82, *p* < 0.0001). For functional species and individuals, sequential reduction of phylum numbers caused a linear reduction in functional species by an average of 7% (Pearson’s correlation *r*^2^ = 0.97, *p* < 0.0001) and individuals by 17% (Pearson’s correlation *r*^2^ = 0.98, *p* < 0.0001) for each step of simulated species loss.

### Stable Microbial Functional Composition Despite Taxonomic Variation

**FIGURE 3:**
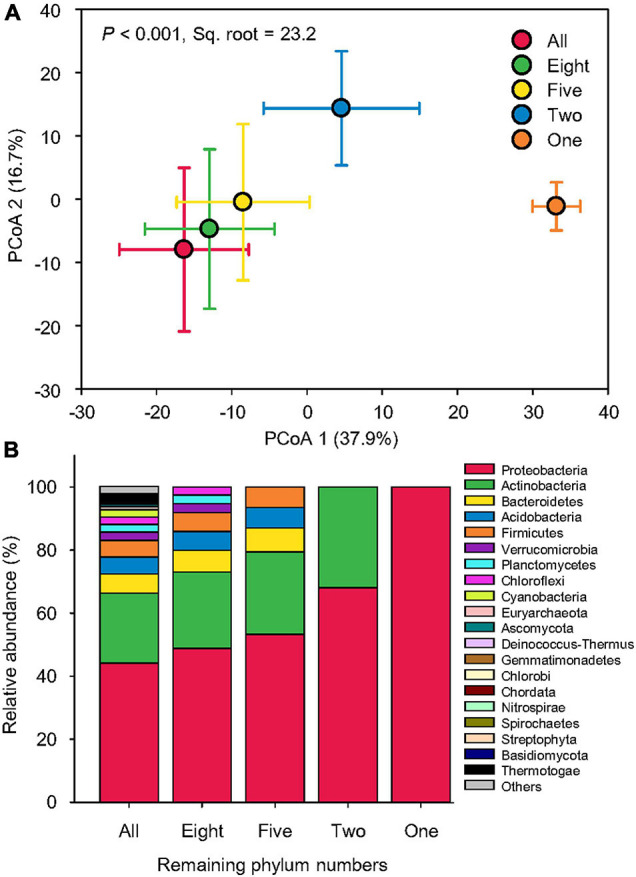
Dramatic taxonomic variation. **(A)** Principal coordinate analysis (PCoA) showing the beta-diversity of taxonomic compositions at the genus level affected by sequential species loss. The error bars represent the standard deviation of data ranges. Variation explained by two principal coordinate dimensions is given in parentheses by percentage. *p* values and Sq. root of PERMANOVA are also given. **(B)** Relative abundance of dominant taxonomic compositions at the phylum level (mean > 0.2%) affected by sequential species loss.

Our simulated species loss resulted in a dramatic shift in beta-diversity of taxonomy. The Bray–Curtis pairwise similarity of taxonomy among samples gradually increased from an average of 66.1 to 74.6%, indicating a trend of community simplification. Hence, the taxonomic compositions of only one phylum, Proteobacteria, revealed the greatest pairwise similarity (Supplementary Figure 1). PCoA showed that the taxonomic structure became more and more distinct from that of all 75 phyla as more phyla were removed (Figure 3A), probably due to the reduction of taxonomic diversity (Figure 3B). The taxonomic compositions in two phyla (Proteobacteria and Actinobacteria; pair-wise pseudo-t = 19.4–27.8, *p* < 0.001) and one phylum (Proteobacteria; pair-wise pseudo-t = 39.7–46.2, *p* < 0.001) were most distinct from others (Supplementary Table 2). PERMANOVA indicated that simulated species loss overall caused 23.2% of beta-diversity variation in taxonomy (pseudo-F = 824.9, *p* < 0.001; Supplementary Table 2).

**FIGURE 4:**
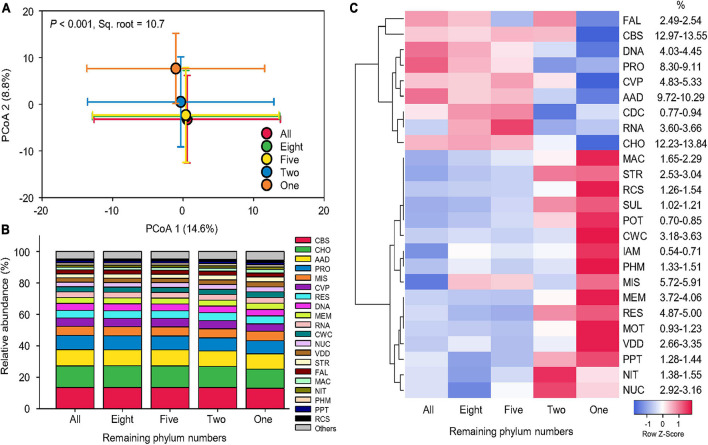
Stable functional structure. **(A)** Principal coordinate analysis (PCoA) showing the beta-diversity of functional profiles at the genus level affected by sequential species loss. The error bars represent the standard deviation of data ranges. Variation explained by two principal coordinate dimensions is given in parentheses by percentage. *p* values and Sq. root of PERMANOVA are also given. **(B)** Relative abundance of dominant functional categories at level 1 (mean > 1%) affected by sequential species loss. **(C)** Heatmaps showing normalized relative abundance of dominant functional categories at level 1 (mean > 1%) affected by sequential species loss. Dendrograms of hierarchical cluster analysis grouping functions are shown. AAD, amino acids and derivatives; CBS, clustering-based subsystems; CDC, cell division and cell cycle; CHO, carbohydrates; CVP, cofactors, vitamins, prosthetic groups, pigments; CWC, cell wall and capsule; DNA, DNA metabolism; FAL, fatty acids, lipids, and isoprenoids; IAM, iron acquisition and metabolism; MAC, metabolism of aromatic compounds; MEM, membrane transport; MIS, miscellaneous; MOT, motility and chemotaxis; NIT, nitrogen metabolism; NUC, nucleosides and nucleotides; PHM, phosphorus metabolism; POT, potassium metabolism; PPT, phages, prophages, transposable elements, plasmids; PRO, protein metabolism; RCS, regulation and cell signaling; RES, respiration; RNA, RNA metabolism; STR, stress response; SUL, sulfur metabolism; and VDD, virulence, disease, and defense.

Compared to taxonomy, the beta-diversity of function was less influenced by our simulated species loss. The Bray–Curtis pairwise similarity of function (54.0–55.4% on average) was not significantly affected by a series of phylum number reductions (Supplementary Figure 1). PCoA showed that the five functional profiles mostly overlapped with each other, although that of one remaining phylum (Proteobacteria) was separated from the others (Figure 4A). PERMANOVA indicated that simulated species loss in general could only explain 10.7% of beta-diversity variation of functional profiles (pseudo-F = 99.3, *p* < 0.001; Supplementary Table 2), considerably less significant than taxonomy, in which species loss treatments could explain 23.2% of beta-diversity variation in taxonomic composition (pseudo-F = 824.9, *p* < 0.001). Pair-wise tests also confirmed that the statistical significance between different levels of species loss was lower in functional profiles (pseudo-t = 6.2–14.6, *p* < 0.001) than taxonomic compositions (pseudo-t = 11.1–46.2, *p* < 0.001; Supplementary Table 2).

Despite significant reduction in species and individuals, the relative abundance of dominant functional categories remained stable across different levels of species loss (Figure 4B). To inspect the slight changes in functional structures induced by the simulated species loss, a heat map was used to show the normalized relative abundance of dominant functional categories at level 1 (Figure 4C). When phyla were sequentially removed from the datasets, broad-scale metabolism functions with higher relative abundance began to decrease. These included clustering-based subsystems, carbohydrates (CHO), amino acids and derivatives, protein metabolism, cofactors, vitamins, prosthetic groups, pigments, DNA metabolism, and RNA metabolism (RNA), which generally decreased by 0.4–1.6% on average. The opposite trend was found in typical nutrient-cycling functions of iron acquisition and metabolism, nitrogen metabolism, phosphorus metabolism (PHM), potassium metabolism, and sulfur metabolism, with an average increase of 0.2%, while stress-response functions, such as cell wall and capsule, metabolism of aromatic compounds, membrane transport, motility and chemotaxis, nucleosides and nucleotides, regulation and cell signaling, stress response (STR), and virulence, disease, and defense, which increased by 0.4% on average. In summary, the variation in the relative abundance of functional categories was limited and function-specific, leading to stable functional structures across different levels of species loss.

**FIGURE 5:**
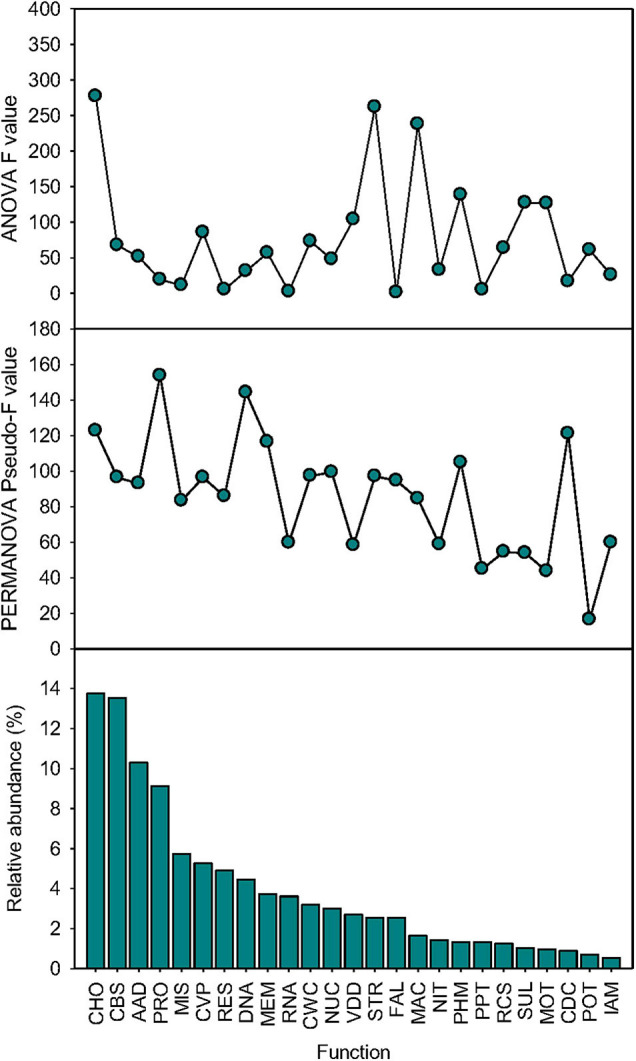
Function-specific effects. The ANOVA statistical significance (*F* value), the PERMANOVA statistical significance (pseudo-*F* value), and relative abundance of 25 dominant functional categories at level 1 (mean > 0.5%) influenced by sequential species loss. AAD, amino acids and derivatives; CBS, clustering-based subsystems; CDC, cell division and cell cycle; CHO, carbohydrates; CVP, cofactors, vitamins, prosthetic groups, pigments; CWC, cell wall and capsule; DNA, DNA metabolism; FAL, fatty acids, lipids, and isoprenoids; IAM, iron acquisition and metabolism; MAC, metabolism of aromatic compounds; MEM, membrane transport; MIS, miscellaneous; MOT, motility and chemotaxis; NIT, nitrogen metabolism; NUC, nucleosides and nucleotides; PHM, phosphorus metabolism; POT, potassium metabolism; PPT, phages, prophages, transposable elements, plasmids; PRO, protein metabolism; RCS, regulation and cell signaling; RES, respiration; RNA, RNA metabolism; STR, stress response; SUL, sulfur metabolism; and VDD, virulence, disease, and defense.

The effects of simulated species loss on the relative abundance and beta-diversity of 25 dominant functions were function-specific (Figure 5). ANOVA showed that the statistical significance (*F* value) of the effect of simulated species loss varied among functional categories, which was independent on their relative abundance. The most affected functions were CHO, STR, and PHM (Supplementary Table 3). When functional composition was renormalized by the relative abundance of each gene at function levels within each functional category at level 1, PERMANOVA revealed that the responses of renormalized functional beta-diversity depended on relative abundance, with the statistical significance (pseudo-*F* value) of dominant functions positively associated with their relative abundance (Pearson’s correlation *r* = 0.51, *p* < 0.001, *n* = 25; Supplementary Table 4). However, certain exceptions existed as the beta-diversity of CDC (pseudo-F = 97.2, *p* < 0.001) and PHM (pseudo-F = 104.8, *p* < 0.001) was significantly shifted by simulated species loss, which only accounted for lower proportions of 0.9 and 1.3%, respectively (Supplementary Table 4).

### Decoupling Co-occurrence Networks of Taxonomy and Function in Response to Species Loss

**FIGURE 6:**
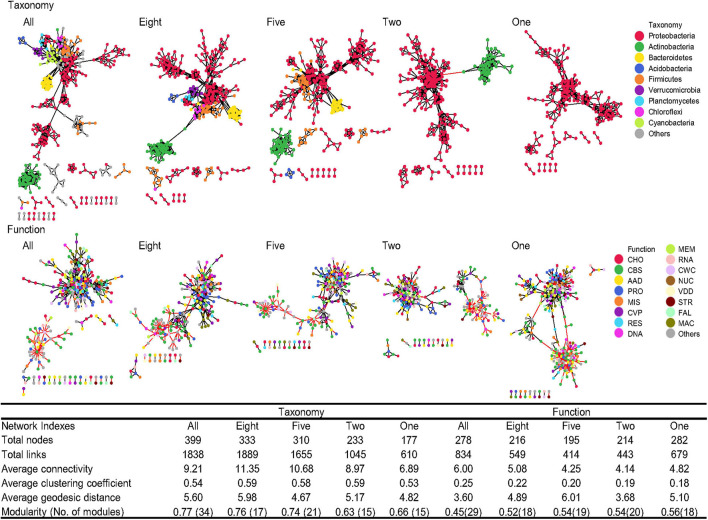
Co-occurrence networks. Network graphs of taxonomic compositions at the genus level (Taxonomy) and functional categories at level 3 (Function) affected by sequential species loss. Node color represents classification of taxonomic compositions at phylum levels and functional categories at level 1. A black edge indicated a positive interaction between two nodes, while a red edge indicated a negative interaction. The summary of key network indexes is given in the table. AAD, amino acids and derivatives; CBS, clustering-based subsystems; CHO, carbohydrates; CVP, cofactors, vitamins, prosthetic groups, pigments; CWC, cell wall and capsule; DNA, DNA metabolism; FAL, fatty acids, lipids, and isoprenoids; MAC, metabolism of aromatic compounds; MEM, membrane transport; MIS, miscellaneous; NUC, nucleosides and nucleotides; PRO, protein metabolism; RES, respiration; RNA, RNA metabolism; STR, stress response; and VDD, virulence, disease, and defense.

Generally, when all 75 phyla were present, the microbial taxonomic network had greater numbers of total nodes, total links, average connectivity, average clustering coefficient, average geodesic distance, and modularity than the functional network (Figure 6), indicating that microbial taxonomic compositions interact more intensely than functional categories, with most of the taxonomic interactions occurring within the same phylum (module), such as major phyla of Proteobacteria, Actinobacteria, and Bacteroidetes. In particular, 15% of the taxonomic links were negative, while functional networks had 29% negative links.

Simulated species loss caused a significant decline in the size and complexity of taxonomic networks. The number of nodes decreased linearly with simulated species loss (Pearson’s correlation *r*^2^ = 0.95). The number of links began to decline when the remaining phyla were less than eight, with negative links reduced to 0%. Overall, the number of nodes and links reduced by 56 and 67%, respectively, and the average connectivity decreased by 25% when the number of phyla was reduced from 75 to one. Since most interactions were within modules of similar species, phylum reduction also reduced the network modularity. Despite the shrinkage of taxonomic networks, functional networks showed no consistent change in response to simulated species loss (Figure 6). The number of nodes and links and the average connectivity were reduced when species loss began, but rose again when the remaining phylum decreased to only Proteobacteria. The proportion of negative links was stabilized in the range of 26–35% across different levels of species loss. With lower modularity compared to taxonomic networks, the interaction of functional networks was present across different functional categories.

## Discussion

Forecasting the changes in microbial functional in case of realistic extinction scenarios in terrestrial ecosystems is important for biodiversity conservation and management when confronting global environmental perturbations (Peter et al., 2011; Philippot et al., 2013; Delgado-Baquerizo et al., 2016a). Most insights into the evaluation of ecosystem functioning in response to biodiversity decline are based on animal and plant communities (Chen et al., 2020), but microbial communities differ fundamentally from macroorganisms due to their high diversity and physiological versatility (Peter et al., 2011). In this study, we clearly showed that a relatively stable functional profile calculated by relative abundance could be maintained in face of dramatic species decline in microbial communities (Figure 4B), as microbial communities have high taxonomic variability but stable functional structure (Louca et al., 2016). It implies a high extent of functional redundancy in the soil microbial community across the globe. It should be noted that while removing phyla may result in a loss of genera, it does not necessarily lead to the loss of function unless the function concerned happens to be restricted to the removed phyla. Therefore, the functions tested in our study were based on the relative abundance of more than twelve thousands of specific genes, which provided the details of functional composition with resolution finer to function levels. Considering the functions that happen to be restricted to a specific phylum, we can identify the relationship of the loss of certain phyla and specific functions.

Yet, the degree of functional redundancy in soil microbes really depends on the levels of manipulated variation in community diversity or the strength of disturbances. Using a dilution-to-extinction approach, some studies have shown that the loss in microbial diversity significantly affects functioning, such as microbial respiration, carbon decomposition, and nitrogen cycling (Peter et al., 2011; Philippot et al., 2013; Delgado-Baquerizo et al., 2016a). In our simulation, only 53 and 54% of the total number of individuals in taxonomy and function were removed from the datasets, respectively (Figure 2), which were much less than the dilution-to-extinction approach that induced reductions by orders of magnitudes. However, all these studies of microbial species loss simulation focused on random extinction scenarios, as it is experimentally impossible to directly remove certain microbial groups sequentially. In reality, extinction risk is typically high for rare species with small populations (Solan et al., 2004), because they are more vulnerable to environmental perturbations. If the extinction direction is random, we could simply attribute the stable functional structures to proportionally declined functional abundances. However, we used a biodiversity loss approach that reflects a more realistic scenario by removing less-common species first, so that highly abundant bacterial phyla, such as Proteobacteria and Actinobacteria, remained till the end to represent the “winning” survivors. Nevertheless, we found a nearly constant functional structure in the simplified communities compared to diverse ones with Archaea, Bacteria, and Eukaryota. It should be noted that in this study, the order of taxon removal was based on the relative abundance at the phylum level. Thus, future studies could examine the effect of ranking the abundance at the genus level or even applying random species loss as comparison for similar analyses.

Interestingly, species loss did not reduce microbial taxonomic species and individuals in the same magnitude. For example, the first step of reduction to eight dominant bacterial phyla reduced nearly half of the taxonomic species but only caused a reduction of taxonomic individuals by 9%, suggesting that the removed 77 microbial phyla, including Archaea, Bacteria, and Eukaryota, included more diverse species but less relative abundance compared to the rest of the phylum-removing steps. On the contrary, sequential reduction of phylum numbers caused a linear reduction of both functional species and individuals, suggesting that although sequential species loss may cause an unequal decline of taxonomic species, the functional abundance was more proportionately reduced by each step of phylum number reduction. However, a steady functional response to species loss was calculated by relative abundance, so a significant reduction of total functional abundances, such as microbial activity, can cause serious damage to ecosystems if functional richness is lost together with microbial species.

In addition, the functions measured in the previous studies were limited to simple broad-scale functions, which cannot provide a broad and detailed picture of multiple functions performed by different microbes, particularly when high microbial diversity in terrestrial ecosystems was considered. By comparing metagenomes of microbial communities, tens of thousands of functions can be evaluated at the same time, enabling deep assessment of higher level of functional diversity in our simulation. More importantly, the single or limited microbiomes tested in previous studies are too restricted to elucidate the relationship between taxonomic and functional diversities in a global perspective. Our results were simulated based on worldwide soil metagenomic datasets, covering various biomes, and hence can represent the diverse traits of soil microorganisms and strongly support that potential functional profile can be prospectively decoupled from taxonomic composition under certain circumstances, as has been suggested in a previous study (Louca et al., 2018). It is often assumed that genome streamlining (Morris et al., 2012) and horizontal gene transfer (David and Alm, 2011), common in prokaryotic populations, have contributed to the functional similarity among distinct taxa. Some studies have observed a linear relationship between functional and taxonomic diversities, suggesting a somewhat dependency of microbial functional profiles on taxonomic compositions (Fierer et al., 2012b, 2013; Leff et al., 2015). Our study differs from these experiments in that the RefSeq database was used for taxonomic assignment, whose diversity was greater than traditional ribosomal RNA databases commonly applied in previous research. Thus, we found that the taxonomic dissimilarity, pseudo-*F* value, was one order of magnitude larger than the function, leading to a decoupling of function from taxonomy in terrestrial microbial community as functional diversity remains relatively more stable.

It is often assumed that microbial diversity reduction in natural soils would affect specialized microbial functions, such as nutrient-cycling processes, more significantly than broad-scale metabolic functions (Yin et al., 2000; Rousk et al., 2009; Banerjee et al., 2016). We did not evidence that microbial functions of relatively lower abundances responded more sensitively to species loss, as the statistical significance (*F* value) of ANOVA was not dependent on the relative abundance of each function (Figure 5). Interestingly, we observed an opposite response that the broad-scale metabolisms conducted by a wide range of soil microbes decreased with community simplification, while typical nutrient-cycling functions relatively increased thereafter. These trends were mainly due to the order by which microbial community were reduced, as the remaining “winners,” Proteobacteria and Actinobacteria, are often considered the major regulators of terrestrial nutrient cycles (Dai et al., 2018, 2020). For example, certain sulfate- and iron-reducing bacteria, *Desulfovibrio* and *Desulfobulbus*, are Deltaproteobacteria (Muyzer and Stams, 2008), and some bacteria conducting N cycling, such as ammonia oxidizers (Stephen et al., 1996) and rhizobia for N fixation (Moulin et al., 2001), mainly belong to Alphaproteobacteria or Betaproteobacteria. Due to the limitation of the functional datasets that are primarily based on metabolic reconstructions of bacterial genomes (Glass et al., 2010), we may underestimate the contribution of those microbes other than the major remaining bacterial phyla to soil nutrient cycling, since archaea and fungi are discovered to play an important role in driving terrestrial biogeochemical cycles (Offre et al., 2013), especially carbon and nitrogen functions (Read and Perez-Moreno, 2003; Veresoglou et al., 2012), such as methane production (Evans et al., 2019), and ammonia oxidation (Stahl and De La Torre, 2012).

When examining the functional profile of each functional category at level 1, which was detailed to specific function levels, we found that the variation of the beta-diversity of functions responding to species loss positively correlated with their relative abundance, suggesting that the simulated reduction of microbial species potentially affects the composition of the high-abundance functional categories more significantly than those of lower abundance. Therefore, microbial homogenization caused different function-specific effects on the beta-diversity of functional profiles between higher- and lower-abundance functions. However, these differences may be merely because functions with higher abundance contained more categories of specific genes, which had higher relative abundance than those of less abundant functions. Thus, the variation may be more significant for the functional composition of more abundant functions based on the calculation of the relative abundance of each gene at function levels. When we calculated the relative abundance of each functional category at level 1, the statistical significance was independent on their relative abundance. Thus, it is unlikely to draw a direct conclusion that less abundant functions react to simulated species loss less significantly than more abundant functions. Future studies evaluating the functional changes caused by species loss should emphasize on finer levels of functional resolution to avoid missing the variation of functional profiles occurring at finer levels.

Due to the relatively stable functional structure, the interaction patterns of functional species were also similar across diversity decline levels without any notable loss of certain functional categories (Figure 6). Simulated species loss caused a significant decline in the numbers of total nodes and negative links, suggesting that when community simplification makes the remaining microbes more uniform and similar to each other, the microbial interactions become mostly cooperative (Faust and Raes, 2012). Thus, soil microbes under community simplification tended to respond to the environment in a similar fashion, while distinct microorganisms before community simplification competitively interact with each other, reflecting regulatory or suppression relationships (Ma et al., 2018). However, the removal of certain phyla may reduce the numbers of species and individuals but did not affect the functional beta-diversity based on the relative abundance, and thus did not affect the overall interaction patterns in functions. The negative links also remained stable across different levels of species loss, showing that species loss did not make functional networks more facilitative or inhibitive. These findings support potentially decoupling responses of co-occurring patterns in taxonomy and function under simulated species loss. Future research, using a series of diversity reductions, to evaluate specific functions should also focus on the co-occurring patterns of multiple functions, which may help better elucidate the influence of species diversity on ecosystem functioning.

## Conclusion

For the first time, we present a comparison of five levels of soil microbial diversities of taxonomy and function responding to biodiversity loss based on global soil metagenomes across diverse biomes. We reveal that the relative abundance of microbial function can remain stable despite that taxonomic species decrease dramatically, leading to biotic homogenization but functional stability. Thus, biodiversity loss continuously shrinks the size and complexity of taxonomic interaction networks but did not affect the overall interaction patterns in functions. Sequential species loss also caused the dominant functions to change from broad-scale metabolism to typical nutrient-cycling. This study has potentially far-reaching implications for biodiversity conservation in species-rich terrestrial ecosystems that have high levels of microbial physiological versatility in the face of realistic species loss scenario.

## Data Availability Statement

The datasets presented in this study can be found in online repositories. The names of the repository/repositories and accession number(s) can be found in the article/Supplementary Material.

## Author Contributions

HC and ZY conceived the study, performed data analysis, interpreted the results, and drafted the manuscript. HC, ZY, and CC secured the research funding. KM, YH, and CC critically assessed and interpreted the findings. All authors discussed the results, commented on and edited the manuscript, revised for important intellectual content, and approved the manuscript.

## Conflict of Interest

The authors declare that the research was conducted in the absence of any commercial or financial relationships that could be construed as a potential conflict of interest.

## Publisher’s Note

All claims expressed in this article are solely those of the authors and do not necessarily represent those of their affiliated organizations, or those of the publisher, the editors and the reviewers. Any product that may be evaluated in this article, or claim that may be made by its manufacturer, is not guaranteed or endorsed by the publisher.

## Footnotes

1
http://ieg4.rccc.ou.edu/MENA/
