# Supplementary material for: Stable Soil Microbial Functional Structure Responding to Biodiversity Loss Based on Metagenomic Evidences
Source: Front Microbiol. 2021 Oct 7;12:716764. doi: 10.3389/fmicb.2021.716764 (PMC8529109; doi:10.3389/fmicb.2021.716764)

**Supplementary Table 1.** **Information of MG-RAST soil metagenomes.** Data are extracted from publications used in this study including study ID, MG-RAST ID, publication, bp, reads, latitude and longitude.

| Study ID | MG-RAST ID | Publication | bp | Reads | LAT | LONG |
| --- | --- | --- | --- | --- | --- | --- |
| 82200 | 4763079 | Albright 2018 | 2365007462 | 14620881 | 35.58 | -79.05 |
| 82200 | 4763080 | Albright 2018 | 2762419447 | 16198823 | 35.58 | -79.05 |
| 82200 | 4763081 | Albright 2018 | 2554900306 | 15212164 | 35.58 | -79.05 |
| 82200 | 4763082 | Albright 2018 | 3395785485 | 22026817 | 35.58 | -79.05 |
| 82200 | 4763083 | Albright 2018 | 2315264262 | 13831020 | 35.58 | -79.05 |
| 82200 | 4763084 | Albright 2018 | 3806860607 | 24683512 | 35.58 | -79.05 |
| 82200 | 4763088 | Albright 2018 | 2957904216 | 17773229 | 35.58 | -79.05 |
| 82200 | 4763090 | Albright 2018 | 2786742080 | 16716482 | 35.58 | -79.05 |
| 82200 | 4763091 | Albright 2018 | 4190163866 | 27186304 | 35.58 | -79.05 |
| 82200 | 4763092 | Albright 2018 | 2726497395 | 16198794 | 35.58 | -79.05 |
| 82200 | 4763093 | Albright 2018 | 3669306904 | 23801355 | 35.58 | -79.05 |
| 82200 | 4763094 | Albright 2018 | 3605065426 | 23324417 | 35.58 | -79.05 |
| 82200 | 4763096 | Albright 2018 | 2705796539 | 16239547 | 35.58 | -79.05 |
| 82200 | 4763097 | Albright 2018 | 2713107142 | 16151462 | 35.58 | -79.05 |
| 82200 | 4763099 | Albright 2018 | 2722758068 | 16571500 | 35.58 | -79.05 |
| 82200 | 4763100 | Albright 2018 | 2739462201 | 16384625 | 35.58 | -79.05 |
| 82200 | 4763104 | Albright 2018 | 2526579015 | 15281768 | 35.58 | -79.05 |
| 82200 | 4763105 | Albright 2018 | 2775295921 | 16712510 | 35.58 | -79.05 |
| 82200 | 4763107 | Albright 2018 | 2347740643 | 13921186 | 35.58 | -79.05 |
| 82200 | 4763108 | Albright 2018 | 4140914155 | 26861211 | 35.58 | -79.05 |
| 82200 | 4763109 | Albright 2018 | 2417916234 | 14287037 | 35.58 | -79.05 |
| 82200 | 4763111 | Albright 2018 | 2694326254 | 16046857 | 35.58 | -79.05 |
| 82200 | 4763113 | Albright 2018 | 4074588860 | 26390952 | 35.58 | -79.05 |
| 82200 | 4763114 | Albright 2018 | 3068659770 | 19804688 | 35.58 | -79.05 |
| 134 | 4451033 | Andreote 2012 | 58801025 | 249993 | -23.90 | -46.21 |
| 134 | 4451034 | Andreote 2012 | 55077381 | 231233 | -23.90 | -45.25 |
| 134 | 4451035 | Andreote 2012 | 53292298 | 214921 | -25.08 | -45.25 |
| 134 | 4451036 | Andreote 2012 | 48522914 | 217605 | -25.08 | -47.95 |
| 12344 | 4615008 | Argiroff 2017 | 3072781180 | 20762035 | 43.35 | -84.03 |
| 12344 | 4615009 | Argiroff 2017 | 2843747332 | 19214509 | 43.35 | -84.03 |
| 12344 | 4615010 | Argiroff 2017 | 2788642492 | 18842179 | 43.35 | -84.03 |
| 12344 | 4615011 | Argiroff 2017 | 2706666180 | 18288285 | 43.36 | -84.01 |
| 12344 | 4615012 | Argiroff 2017 | 2163835036 | 14620507 | 43.36 | -84.01 |
| 12344 | 4615013 | Argiroff 2017 | 2506049220 | 16932765 | 43.36 | -84.01 |
| 12344 | 4615014 | Argiroff 2017 | 2273464556 | 15361247 | 43.36 | -84.04 |
| 12344 | 4615015 | Argiroff 2017 | 2360950612 | 15952369 | 43.36 | -84.04 |
| 12344 | 4615016 | Argiroff 2017 | 3816698592 | 25788504 | 43.36 | -84.04 |
| 2592 | 4509403 | Bach 2018 | 20524028604 | 203208204 | 41.92 | -93.75 |
| 2592 | 4509407 | Bach 2018 | 8778023120 | 86911120 | 41.92 | -93.75 |
| 13154 | 4625855 | Bell 2016 | 1792387478 | 7584499 | 45.68 | -73.44 |
| 13154 | 4626132 | Bell 2016 | 2211693541 | 9521307 | 45.68 | -73.44 |
| 13154 | 4626133 | Bell 2016 | 1894297276 | 8054077 | 45.68 | -73.44 |
| 13154 | 4626134 | Bell 2016 | 1843515599 | 8038689 | 45.68 | -73.44 |
| 13154 | 4626327 | Bell 2016 | 1668816265 | 7168268 | 45.68 | -73.44 |
| 13154 | 4626321 | Bell 2016 | 2248856746 | 10285623 | 45.68 | -73.44 |
| 13154 | 4626135 | Bell 2016 | 2265745719 | 10257340 | 45.68 | -73.44 |
| 13154 | 4626136 | Bell 2016 | 2038907926 | 9069615 | 45.68 | -73.44 |
| 13154 | 4626322 | Bell 2016 | 2325615329 | 10295352 | 45.68 | -73.44 |
| 13154 | 4626323 | Bell 2016 | 2086123634 | 9320516 | 45.68 | -73.44 |
| 13154 | 4626137 | Bell 2016 | 2318619150 | 10756113 | 45.68 | -73.44 |
| 13154 | 4626324 | Bell 2016 | 3290051684 | 14486708 | 45.68 | -73.44 |
| 13154 | 4625856 | Bell 2016 | 1876251010 | 8034402 | 45.68 | -73.44 |
| 13154 | 4626325 | Bell 2016 | 2075596630 | 9002467 | 45.68 | -73.44 |
| 13154 | 4626328 | Bell 2016 | 2072665418 | 9015838 | 45.68 | -73.44 |
| 13154 | 4626138 | Bell 2016 | 1702467699 | 7230891 | 45.68 | -73.44 |
| 13154 | 4626139 | Bell 2016 | 1763351463 | 7505498 | 45.68 | -73.44 |
| 13154 | 4626140 | Bell 2016 | 2523424544 | 11363851 | 45.68 | -73.44 |
| 2205 | 4502923 | Chang 2017 | 6208794000 | 62087940 | 38.20 | -89.63 |
| 2205 | 4502929 | Chang 2017 | 9012045900 | 90120459 | 38.20 | -89.63 |
| 2205 | 4502930 | Chang 2017 | 12440446700 | 124404467 | 39.38 | -90.32 |
| 2205 | 4502931 | Chang 2017 | 9384932000 | 93849320 | 39.38 | -90.32 |
| 2205 | 4502932 | Chang 2017 | 10633324800 | 106333248 | 39.38 | -90.32 |
| 2205 | 4502933 | Chang 2017 | 6744249700 | 67442497 | 39.38 | -90.32 |
| 2205 | 4502924 | Chang 2017 | 9486724800 | 94867248 | 39.59 | -89.76 |
| 2205 | 4502925 | Chang 2017 | 8934148200 | 89341482 | 40.22 | -88.47 |
| 2205 | 4502926 | Chang 2017 | 9757607000 | 97576070 | 40.22 | -88.47 |
| 2205 | 4502927 | Chang 2017 | 11110850800 | 111108508 | 40.08 | -88.22 |
| 2205 | 4502928 | Chang 2017 | 8122153300 | 81221533 | 40.08 | -88.22 |
| 2205 | 4502934 | Chang 2017 | 6394801600 | 63948016 | 39.60 | -89.76 |
| 2205 | 4502935 | Chang 2017 | 10060714700 | 100607147 | 39.60 | -89.76 |
| 22000 | 4735485 | Chen 2019 | 3481656415 | 10932585 | 35.93 | -84.39 |
| 22000 | 4735486 | Chen 2019 | 3349141456 | 10314339 | 35.93 | -84.39 |
| 22000 | 4735487 | Chen 2019 | 3833575602 | 11652583 | 35.93 | -84.39 |
| 22000 | 4735489 | Chen 2019 | 3266669439 | 10069906 | 35.93 | -84.39 |
| 18393 | 4702982 | Choi 2017 | 251762454 | 1416604 | 37.46 | 126.95 |
| 18393 | 4702995 | Choi 2017 | 295991344 | 1644333 | 37.46 | 126.95 |
| 18393 | 4702971 | Choi 2017 | 263241389 | 1457569 | 37.46 | 126.95 |
| 18393 | 4702989 | Choi 2017 | 268132713 | 1481176 | 37.46 | 126.95 |
| 18393 | 4702980 | Choi 2017 | 334106633 | 1848889 | 37.46 | 126.95 |
| 18393 | 4702967 | Choi 2017 | 570977883 | 3130138 | 37.46 | 126.95 |
| 18393 | 4702972 | Choi 2017 | 759134203 | 4209432 | 37.46 | 126.95 |
| 18393 | 4702984 | Choi 2017 | 1522527691 | 8297494 | 37.46 | 126.95 |
| 18393 | 4702981 | Choi 2017 | 2095879179 | 11428311 | 37.46 | 126.95 |
| 18393 | 4702994 | Choi 2017 | 838829333 | 4584694 | 37.46 | 126.95 |
| 18393 | 4702973 | Choi 2017 | 514420404 | 2823441 | 37.46 | 126.95 |
| 18393 | 4702993 | Choi 2017 | 595898657 | 3301223 | 37.46 | 126.95 |
| 18393 | 4702990 | Choi 2017 | 401251633 | 2208832 | 37.46 | 126.95 |
| 18393 | 4702979 | Choi 2017 | 372902610 | 2056981 | 37.46 | 126.95 |
| 18393 | 4702969 | Choi 2017 | 286633404 | 1631831 | 37.46 | 126.95 |
| 18393 | 4702974 | Choi 2017 | 1382755860 | 7623170 | 37.46 | 126.95 |
| 18393 | 4702991 | Choi 2017 | 726420143 | 3941838 | 37.46 | 126.95 |
| 18393 | 4702976 | Choi 2017 | 681454104 | 3767325 | 37.46 | 126.95 |
| 18393 | 4702992 | Choi 2017 | 524935120 | 2872536 | 37.46 | 126.95 |
| 18393 | 4702983 | Choi 2017 | 379698417 | 2069903 | 37.46 | 126.95 |
| 18393 | 4702988 | Choi 2017 | 458323520 | 2513034 | 37.46 | 126.95 |
| 18393 | 4702987 | Choi 2017 | 393816984 | 2158172 | 37.46 | 126.95 |
| 18393 | 4702985 | Choi 2017 | 425897545 | 2339629 | 37.46 | 126.95 |
| 18393 | 4702966 | Choi 2017 | 584082373 | 3224188 | 37.46 | 126.95 |
| 18393 | 4702978 | Choi 2017 | 516699190 | 2860404 | 37.46 | 126.95 |
| 18393 | 4702986 | Choi 2017 | 370497677 | 2039288 | 37.46 | 126.95 |
| 18393 | 4702977 | Choi 2017 | 845842384 | 4697108 | 37.46 | 126.95 |
| 18393 | 4702968 | Choi 2017 | 266506149 | 1466354 | 37.46 | 126.95 |
| 18393 | 4702975 | Choi 2017 | 344102058 | 1901086 | 37.46 | 126.95 |
| 18393 | 4702970 | Choi 2017 | 496934737 | 2748139 | 37.46 | 126.95 |
| 18857 | 4707563 | Choi 2017 | 572161561 | 3204375 | 37.46 | 126.95 |
| 18857 | 4707570 | Choi 2017 | 575197473 | 3114726 | 37.46 | 126.95 |
| 18857 | 4707583 | Choi 2017 | 554356262 | 3093279 | 37.46 | 126.95 |
| 18857 | 4707561 | Choi 2017 | 545495218 | 3018065 | 37.46 | 126.95 |
| 18857 | 4707558 | Choi 2017 | 829525514 | 4549698 | 37.46 | 126.95 |
| 18857 | 4707571 | Choi 2017 | 766439886 | 4209214 | 37.46 | 126.95 |
| 18857 | 4707568 | Choi 2017 | 676225075 | 3762861 | 37.46 | 126.95 |
| 18857 | 4707585 | Choi 2017 | 643579358 | 3497651 | 37.46 | 126.95 |
| 18857 | 4707580 | Choi 2017 | 650301895 | 3553533 | 37.46 | 126.95 |
| 18857 | 4707586 | Choi 2017 | 715574780 | 3915994 | 37.46 | 126.95 |
| 18857 | 4707562 | Choi 2017 | 729354993 | 4063798 | 37.46 | 126.95 |
| 18857 | 4707566 | Choi 2017 | 601812503 | 3274033 | 37.46 | 126.95 |
| 18857 | 4707564 | Choi 2017 | 570773518 | 3155286 | 37.46 | 126.95 |
| 18857 | 4707582 | Choi 2017 | 552567024 | 3026986 | 37.46 | 126.95 |
| 18857 | 4707575 | Choi 2017 | 731378265 | 4023231 | 37.46 | 126.95 |
| 18857 | 4707573 | Choi 2017 | 756048896 | 4139652 | 37.46 | 126.95 |
| 18857 | 4707567 | Choi 2017 | 566112460 | 3108174 | 37.46 | 126.95 |
| 18857 | 4707565 | Choi 2017 | 634245205 | 3399243 | 37.46 | 126.95 |
| 18857 | 4707576 | Choi 2017 | 580058103 | 3246637 | 37.46 | 126.95 |
| 18857 | 4707579 | Choi 2017 | 691211973 | 3862301 | 37.46 | 126.95 |
| 18857 | 4707560 | Choi 2017 | 696990832 | 3868293 | 37.46 | 126.95 |
| 18857 | 4707581 | Choi 2017 | 652609696 | 3637570 | 37.46 | 126.95 |
| 18857 | 4707574 | Choi 2017 | 538489571 | 2992906 | 37.46 | 126.95 |
| 18857 | 4707589 | Choi 2017 | 691109486 | 3855896 | 37.46 | 126.95 |
| 18857 | 4707569 | Choi 2017 | 747307771 | 4102235 | 37.46 | 126.95 |
| 18857 | 4707559 | Choi 2017 | 750823635 | 4150610 | 37.46 | 126.95 |
| 18857 | 4707584 | Choi 2017 | 582908908 | 3257066 | 37.46 | 126.95 |
| 18857 | 4707577 | Choi 2017 | 609806249 | 3412911 | 37.46 | 126.95 |
| 18857 | 4707572 | Choi 2017 | 652423120 | 3660170 | 37.46 | 126.95 |
| 18857 | 4707588 | Choi 2017 | 813704850 | 4455554 | 37.46 | 126.95 |
| 18857 | 4707587 | Choi 2017 | 801754876 | 4427211 | 37.46 | 126.95 |
| 18857 | 4707578 | Choi 2017 | 627969701 | 3473797 | 37.46 | 126.95 |
| 5588 | 4541641 | Cline 2015 | 7700742394 | 50998294 | 45.40 | -93.20 |
| 5588 | 4539063 | Cline 2015 | 7942507135 | 52599385 | 45.40 | -93.20 |
| 5588 | 4541642 | Cline 2015 | 8791533476 | 58222076 | 45.40 | -93.20 |
| 5588 | 4539064 | Cline 2015 | 7818845383 | 51780433 | 45.40 | -93.20 |
| 5588 | 4541644 | Cline 2015 | 7034330923 | 46584973 | 45.40 | -93.20 |
| 5588 | 4541645 | Cline 2015 | 10745529044 | 71162444 | 45.40 | -93.20 |
| 5588 | 4541646 | Cline 2015 | 11702083089 | 77497239 | 45.40 | -93.20 |
| 5588 | 4541647 | Cline 2015 | 9453732047 | 62607497 | 45.40 | -93.20 |
| 5588 | 4541648 | Cline 2015 | 7567538499 | 50116149 | 45.40 | -93.20 |
| 5588 | 4541649 | Cline 2015 | 8876832923 | 58786973 | 45.40 | -93.20 |
| 5588 | 4541650 | Cline 2015 | 7450633091 | 49341941 | 45.40 | -93.20 |
| 5588 | 4541651 | Cline 2015 | 8731088931 | 57821781 | 45.40 | -93.20 |
| 15600 | 4670122 | Cline 2017 | 7678885748 | 50853548 | 48.30 | -110.66 |
| 15600 | 4670116 | Cline 2017 | 6155685630 | 40766130 | 48.30 | -110.66 |
| 15600 | 4670123 | Cline 2017 | 6942986342 | 45980042 | 43.57 | -110.31 |
| 15600 | 4670120 | Cline 2017 | 7003222356 | 46378956 | 43.57 | -110.31 |
| 15600 | 4670121 | Cline 2017 | 6296889656 | 41701256 | 43.30 | -110.66 |
| 15600 | 4670118 | Cline 2017 | 6105280471 | 40432321 | 43.30 | -110.66 |
| 15600 | 4670119 | Cline 2017 | 4774945103 | 31622153 | 43.51 | -110.71 |
| 15600 | 4670117 | Cline 2017 | 4668384403 | 30916453 | 43.51 | -110.71 |
| 3608 | 4465558 | Cobo-Díaz 2015 | 80126156 | 257697 | 36.96 | -3.46 |
| 3608 | 4465556 | Cobo-Díaz 2015 | 159918596 | 412302 | 36.97 | -3.46 |
| 405 | 4453246 | Delmont 2012 | 358393415 | 976268 | 51.48 | 0.22 |
| 405 | 4453247 | Delmont 2012 | 470523038 | 1094883 | 51.48 | 0.22 |
| 405 | 4453254 | Delmont 2012 | 321342919 | 890966 | 51.48 | 0.22 |
| 405 | 4453407 | Delmont 2012 | 311395302 | 754829 | 51.48 | 0.22 |
| 405 | 4453257 | Delmont 2012 | 391415961 | 946839 | 51.48 | 0.22 |
| 405 | 4453274 | Delmont 2012 | 256246231 | 754185 | 51.48 | 0.22 |
| 405 | 4453433 | Delmont 2012 | 308792456 | 826513 | 51.48 | 0.22 |
| 405 | 4453261 | Delmont 2012 | 465558160 | 1130719 | 51.48 | 0.22 |
| 405 | 4453434 | Delmont 2012 | 433202892 | 1137813 | 51.48 | 0.22 |
| 405 | 4453436 | Delmont 2012 | 343359668 | 919406 | 51.48 | 0.22 |
| 405 | 4453435 | Delmont 2012 | 371406384 | 1068770 | 51.48 | 0.22 |
| 405 | 4453256 | Delmont 2012 | 505500140 | 1135084 | 51.48 | 0.22 |
| 405 | 4453406 | Delmont 2012 | 338504768 | 938860 | 51.48 | 0.22 |
| 8624 | 4558897 | Dini-Andreote 2014 | 2176321831 | 13242159 | 53.49 | 6.22 |
| 8624 | 4558900 | Dini-Andreote 2014 | 3401282325 | 20528012 | 53.49 | 6.22 |
| 8624 | 4558903 | Dini-Andreote 2014 | 1938333700 | 11522239 | 53.49 | 6.22 |
| 8624 | 4558906 | Dini-Andreote 2014 | 2255559508 | 12813819 | 53.49 | 6.22 |
| 8624 | 4558909 | Dini-Andreote 2014 | 2777721432 | 16485482 | 53.49 | 6.22 |
| 8624 | 4558912 | Dini-Andreote 2014 | 2826920802 | 18046584 | 53.49 | 6.22 |
| 8624 | 4558915 | Dini-Andreote 2014 | 3205571588 | 19548727 | 53.49 | 6.22 |
| 8624 | 4558918 | Dini-Andreote 2014 | 1847792318 | 11148206 | 53.49 | 6.22 |
| 8624 | 4558921 | Dini-Andreote 2014 | 2418443284 | 14798632 | 53.49 | 6.22 |
| 8624 | 4558924 | Dini-Andreote 2014 | 2107365646 | 12835938 | 53.49 | 6.22 |
| 8624 | 4558927 | Dini-Andreote 2014 | 2072319370 | 11648006 | 53.49 | 6.22 |
| 8624 | 4558930 | Dini-Andreote 2014 | 1273965121 | 7103663 | 53.49 | 6.22 |
| 8624 | 4558933 | Dini-Andreote 2014 | 437994379 | 2379208 | 53.49 | 6.22 |
| 8624 | 4558936 | Dini-Andreote 2014 | 573508052 | 3150283 | 53.49 | 6.22 |
| 8624 | 4558939 | Dini-Andreote 2014 | 2993511909 | 18105028 | 53.49 | 6.22 |
| 2997 | 4477807 | Fierer 2012 | 920666200 | 9206662 | -12.63 | -71.23 |
| 2997 | 4477872 | Fierer 2012 | 680545600 | 6805456 | 35.38 | -105.93 |
| 2997 | 4477873 | Fierer 2012 | 1112254600 | 11122546 | 34.33 | -106.73 |
| 2997 | 4477805 | Fierer 2012 | 589949700 | 5899497 | 34.90 | -115.65 |
| 2997 | 4477875 | Fierer 2012 | 523535200 | 5235352 | -26.73 | -54.68 |
| 2997 | 4477877 | Fierer 2012 | 640294000 | 6402940 | 34.62 | -81.67 |
| 2997 | 4477899 | Fierer 2012 | 389004400 | 3890044 | 35.97 | -79.08 |
| 2997 | 4477804 | Fierer 2012 | 534883200 | 5348832 | 39.10 | -96.60 |
| 2997 | 4477876 | Fierer 2012 | 654390300 | 6543903 | 64.80 | -148.25 |
| 2997 | 4477874 | Fierer 2012 | 601197100 | 6011971 | 68.63 | -149.58 |
| 2997 | 4477900 | Fierer 2012 | 794708600 | 7947086 | -78.03 | 163.87 |
| 2997 | 4477901 | Fierer 2012 | 545464000 | 5454640 | -77.73 | 162.31 |
| 2997 | 4477902 | Fierer 2012 | 944668400 | 9446684 | -77.61 | 163.25 |
| 2997 | 4477903 | Fierer 2012 | 654368100 | 6543681 | -77.64 | 162.89 |
| 2997 | 4477904 | Fierer 2012 | 1086364600 | 10863646 | -77.53 | 161.70 |
| 2997 | 4477803 | Fierer 2012 | 595168400 | 5951684 | -77.73 | 162.31 |
| 4745 | 4449356 | Fierer 2012 | 16491779 | 49322 | 42.44 | -85.38 |
| 4745 | 4449358 | Fierer 2012 | 30994869 | 90765 | 42.44 | -85.38 |
| 4745 | 4449359 | Fierer 2012 | 21339728 | 62232 | 42.44 | -85.38 |
| 4745 | 4449360 | Fierer 2012 | 29380616 | 84770 | 42.44 | -85.38 |
| 4745 | 4449362 | Fierer 2012 | 30469304 | 88074 | 42.44 | -85.38 |
| 4745 | 4449363 | Fierer 2012 | 23810360 | 69516 | 42.44 | -85.38 |
| 4745 | 4449364 | Fierer 2012 | 22988021 | 65582 | 42.44 | -85.38 |
| 4745 | 4449365 | Fierer 2012 | 13736747 | 39365 | 42.44 | -85.38 |
| 4745 | 4449252 | Fierer 2012 | 36665161 | 92615 | 45.40 | -93.27 |
| 4745 | 4449255 | Fierer 2012 | 32258894 | 80499 | 45.40 | -93.27 |
| 4745 | 4449256 | Fierer 2012 | 35553938 | 87810 | 45.40 | -93.27 |
| 4745 | 4449258 | Fierer 2012 | 26412606 | 66029 | 45.40 | -93.27 |
| 4745 | 4449357 | Fierer 2012 | 25790999 | 64560 | 45.40 | -93.27 |
| 4745 | 4449284 | Fierer 2012 | 30913425 | 77306 | 45.40 | -93.27 |
| 4745 | 4449877 | Fierer 2012 | 34126987 | 84858 | 45.40 | -93.27 |
| 4745 | 4449249 | Fierer 2012 | 23664198 | 59998 | 45.40 | -93.27 |
| 10591 | 4579277 | Fonseca 2018 | 648451865 | 2694917 | -8.38 | -63.63 |
| 10591 | 4579275 | Fonseca 2018 | 413336354 | 1725895 | -8.38 | -63.63 |
| 10591 | 4579276 | Fonseca 2018 | 435889590 | 2116070 | -8.38 | -63.63 |
| 5845 | 4535167 | Fonseca 2018 | 630081836 | 2715594 | -8.38 | -63.63 |
| 5845 | 4535136 | Fonseca 2018 | 296443716 | 1293070 | -8.38 | -63.63 |
| 5845 | 4535204 | Fonseca 2018 | 587773541 | 2610515 | -8.38 | -63.63 |
| 14795 | 4661587 | Fraser 2018 | 1167185047 | 3599493 | -26.06 | 113.73 |
| 14795 | 4661588 | Fraser 2018 | 1197907978 | 3757584 | -26.06 | 113.73 |
| 14795 | 4661589 | Fraser 2018 | 1207337003 | 3841773 | -26.06 | 113.73 |
| 14795 | 4661590 | Fraser 2018 | 1125452740 | 3495614 | -25.77 | 113.72 |
| 14795 | 4661591 | Fraser 2018 | 1197070339 | 3753386 | -25.77 | 113.72 |
| 14795 | 4661592 | Fraser 2018 | 1466828783 | 4493000 | -25.62 | 113.59 |
| 14795 | 4661593 | Fraser 2018 | 1192194650 | 3714494 | -25.82 | 113.83 |
| 14795 | 4661594 | Fraser 2018 | 1167798847 | 3528382 | -25.77 | 113.72 |
| 14795 | 4661595 | Fraser 2018 | 1214340697 | 3776363 | -25.62 | 113.59 |
| 14795 | 4661596 | Fraser 2018 | 1184808271 | 3672117 | -25.86 | 113.78 |
| 14795 | 4661597 | Fraser 2018 | 1224919206 | 3854469 | -25.86 | 113.78 |
| 14795 | 4661598 | Fraser 2018 | 1209795764 | 3786067 | -25.93 | 113.75 |
| 14795 | 4661599 | Fraser 2018 | 1186351414 | 3678190 | -25.93 | 113.75 |
| 14795 | 4661600 | Fraser 2018 | 1233436148 | 3836447 | -25.62 | 113.59 |
| 14795 | 4661601 | Fraser 2018 | 1202976906 | 3755410 | -25.86 | 113.78 |
| 14795 | 4661602 | Fraser 2018 | 1206253769 | 3777840 | -25.93 | 113.75 |
| 14795 | 4661603 | Fraser 2018 | 1214421809 | 3754913 | -25.82 | 113.83 |
| 14795 | 4661604 | Fraser 2018 | 1160096662 | 3634852 | -25.82 | 113.83 |
| 13011 | 4623658 | Gibbons 2017 | 1439771867 | 14255167 | 46.68 | -114.03 |
| 13011 | 4623639 | Gibbons 2017 | 1122131210 | 11110210 | 46.68 | -114.03 |
| 13011 | 4623640 | Gibbons 2017 | 1152978428 | 11415628 | 46.68 | -114.03 |
| 13011 | 4623641 | Gibbons 2017 | 1363452328 | 13499528 | 46.68 | -114.03 |
| 13011 | 4623642 | Gibbons 2017 | 1530428356 | 15152756 | 46.68 | -114.03 |
| 13011 | 4623643 | Gibbons 2017 | 266181258 | 2635458 | 46.68 | -114.03 |
| 13011 | 4623644 | Gibbons 2017 | 3075166291 | 30447191 | 46.68 | -114.03 |
| 13011 | 4623645 | Gibbons 2017 | 1541232730 | 15259730 | 46.68 | -114.03 |
| 13011 | 4623646 | Gibbons 2017 | 2163796730 | 21423730 | 46.68 | -114.03 |
| 13011 | 4623647 | Gibbons 2017 | 1745886404 | 17286004 | 46.68 | -114.03 |
| 13011 | 4623650 | Gibbons 2017 | 2017584484 | 19976084 | 46.68 | -114.03 |
| 13011 | 4623648 | Gibbons 2017 | 1280467698 | 12677898 | 46.68 | -114.03 |
| 13011 | 4623649 | Gibbons 2017 | 2920732746 | 28918146 | 46.68 | -114.03 |
| 13011 | 4623651 | Gibbons 2017 | 1761221638 | 17437838 | 46.68 | -114.03 |
| 13011 | 4623652 | Gibbons 2017 | 1352693101 | 13393001 | 46.68 | -114.03 |
| 13011 | 4623653 | Gibbons 2017 | 1030057085 | 10198585 | 46.68 | -114.03 |
| 13011 | 4623654 | Gibbons 2017 | 3071320514 | 30409114 | 46.68 | -114.03 |
| 13011 | 4623655 | Gibbons 2017 | 2198562041 | 21767941 | 46.68 | -114.03 |
| 13011 | 4623656 | Gibbons 2017 | 920379872 | 9112672 | 46.68 | -114.03 |
| 13011 | 4623657 | Gibbons 2017 | 1703423378 | 16865578 | 46.68 | -114.03 |
| 13011 | 4623677 | Gibbons 2017 | 1698567702 | 16817502 | 46.68 | -114.03 |
| 13011 | 4623659 | Gibbons 2017 | 2050575528 | 20302728 | 46.68 | -114.03 |
| 13011 | 4623660 | Gibbons 2017 | 1082151269 | 10714369 | 46.68 | -114.03 |
| 13011 | 4623661 | Gibbons 2017 | 1636563095 | 16203595 | 46.68 | -114.03 |
| 13011 | 4623664 | Gibbons 2017 | 1391825046 | 13780446 | 46.68 | -114.03 |
| 13011 | 4623662 | Gibbons 2017 | 1775567678 | 17579878 | 46.68 | -114.03 |
| 13011 | 4623663 | Gibbons 2017 | 1945098198 | 19258398 | 46.68 | -114.03 |
| 13011 | 4623665 | Gibbons 2017 | 1058172556 | 10476956 | 46.68 | -114.03 |
| 13011 | 4623666 | Gibbons 2017 | 1441625722 | 14273522 | 46.68 | -114.03 |
| 13011 | 4623667 | Gibbons 2017 | 4063655816 | 40234216 | 46.68 | -114.03 |
| 13011 | 4623668 | Gibbons 2017 | 2176061059 | 21545159 | 46.68 | -114.03 |
| 13011 | 4623669 | Gibbons 2017 | 2357431405 | 23340905 | 46.68 | -114.03 |
| 13011 | 4623670 | Gibbons 2017 | 1419250586 | 14051986 | 46.68 | -114.03 |
| 13011 | 4623671 | Gibbons 2017 | 1802815862 | 17849662 | 46.68 | -114.03 |
| 13011 | 4623672 | Gibbons 2017 | 1610635385 | 15946885 | 46.68 | -114.03 |
| 13011 | 4623674 | Gibbons 2017 | 1160768861 | 11492761 | 46.68 | -114.03 |
| 13011 | 4623673 | Gibbons 2017 | 1986017742 | 19663542 | 46.68 | -114.03 |
| 13011 | 4623675 | Gibbons 2017 | 2254354441 | 22320341 | 46.68 | -114.03 |
| 13011 | 4623676 | Gibbons 2017 | 2477030858 | 24525058 | 46.68 | -114.03 |
| 13011 | 4623678 | Gibbons 2017 | 2655821260 | 26295260 | 46.68 | -114.03 |
| 13011 | 4623679 | Gibbons 2017 | 2705204099 | 26784199 | 46.68 | -114.03 |
| 13011 | 4623680 | Gibbons 2017 | 1530670554 | 15155154 | 46.68 | -114.03 |
| 13011 | 4623681 | Gibbons 2017 | 1543227379 | 15279479 | 46.68 | -114.03 |
| 13011 | 4623682 | Gibbons 2017 | 1556593416 | 15411816 | 46.68 | -114.03 |
| 13011 | 4623683 | Gibbons 2017 | 626477851 | 6202751 | 46.68 | -114.03 |
| 13011 | 4623684 | Gibbons 2017 | 382385697 | 3785997 | 46.68 | -114.03 |
| 13011 | 4623686 | Gibbons 2017 | 1107281281 | 10963181 | 46.68 | -114.03 |
| 13011 | 4623685 | Gibbons 2017 | 2246258079 | 22240179 | 46.68 | -114.03 |
| 13011 | 4623687 | Gibbons 2017 | 425125766 | 4209166 | 46.68 | -114.03 |
| 13011 | 4623688 | Gibbons 2017 | 1664009037 | 16475337 | 46.68 | -114.03 |
| 13011 | 4623689 | Gibbons 2017 | 1505819100 | 14909100 | 46.68 | -114.03 |
| 13011 | 4623690 | Gibbons 2017 | 1760535141 | 17431041 | 46.68 | -114.03 |
| 13011 | 4623694 | Gibbons 2017 | 1606518524 | 15906124 | 46.68 | -114.03 |
| 13011 | 4623691 | Gibbons 2017 | 1260382232 | 12479032 | 46.68 | -114.03 |
| 13011 | 4623692 | Gibbons 2017 | 1205724769 | 11937869 | 46.68 | -114.03 |
| 13011 | 4623693 | Gibbons 2017 | 129101735 | 1278235 | 46.68 | -114.03 |
| 13011 | 4623695 | Gibbons 2017 | 1413505807 | 13995107 | 46.68 | -114.03 |
| 13011 | 4623696 | Gibbons 2017 | 1333402 | 13202 | 46.68 | -114.03 |
| 13011 | 4623697 | Gibbons 2017 | 3061850855 | 30315355 | 46.68 | -114.03 |
| 13011 | 4623698 | Gibbons 2017 | 1977456275 | 19578775 | 46.68 | -114.03 |
| 13011 | 4623699 | Gibbons 2017 | 3162578559 | 31312659 | 46.68 | -114.03 |
| 13011 | 4623700 | Gibbons 2017 | 1248483725 | 12361225 | 46.68 | -114.03 |
| 13011 | 4623701 | Gibbons 2017 | 2655975487 | 26296787 | 46.68 | -114.03 |
| 13011 | 4623702 | Gibbons 2017 | 1273222261 | 12606161 | 46.68 | -114.03 |
| 6787 | 4543770 | Guo 2016 | 2802389196 | 5503592 | 42.39 | -85.37 |
| 6787 | 4543771 | Guo 2016 | 3001168673 | 5944107 | 42.39 | -85.37 |
| 6787 | 4543772 | Guo 2016 | 3519218745 | 7033413 | 42.39 | -85.37 |
| 6787 | 4543773 | Guo 2016 | 3342188659 | 6801453 | 42.39 | -85.37 |
| 6787 | 4543774 | Guo 2016 | 3656016194 | 7248199 | 42.39 | -85.37 |
| 6787 | 4543775 | Guo 2016 | 3239323040 | 6430052 | 42.39 | -85.37 |
| 6787 | 4543776 | Guo 2016 | 3044683146 | 6104071 | 42.39 | -85.37 |
| 6787 | 4543777 | Guo 2016 | 3641445144 | 7748864 | 42.39 | -85.37 |
| 6787 | 4543778 | Guo 2016 | 3595458649 | 7646400 | 42.39 | -85.37 |
| 6787 | 4543779 | Guo 2016 | 3367825461 | 7167505 | 42.39 | -85.37 |
| 6787 | 4543780 | Guo 2016 | 2775431641 | 5981679 | 42.39 | -85.37 |
| 6787 | 4543781 | Guo 2016 | 2932326296 | 6295398 | 42.39 | -85.37 |
| 6787 | 4543782 | Guo 2016 | 2727849635 | 5901128 | 42.39 | -85.37 |
| 6787 | 4543783 | Guo 2016 | 2698799435 | 5730646 | 42.39 | -85.37 |
| 6787 | 4543784 | Guo 2016 | 2832577702 | 6046926 | 42.39 | -85.37 |
| 6787 | 4543785 | Guo 2016 | 2497624874 | 5271946 | 42.39 | -85.37 |
| 6787 | 4543786 | Guo 2016 | 2970568996 | 6362710 | 42.39 | -85.37 |
| 6787 | 4543787 | Guo 2016 | 2889374366 | 6225304 | 42.39 | -85.37 |
| 6787 | 4543788 | Guo 2016 | 2425302998 | 5080839 | 42.39 | -85.37 |
| 6787 | 4543789 | Guo 2016 | 2953911202 | 6309742 | 42.39 | -85.37 |
| 6787 | 4543790 | Guo 2016 | 2662683609 | 5711024 | 42.39 | -85.37 |
| 6368 | 4539514 | Howe 2014 | 7056524217 | 87224214 | 41.77 | -92.96 |
| 6368 | 4539515 | Howe 2014 | 7669345313 | 94610611 | 41.77 | -92.96 |
| 6368 | 4539516 | Howe 2014 | 7610751864 | 94581380 | 41.77 | -92.96 |
| 6368 | 4539517 | Howe 2014 | 7904832499 | 98333698 | 41.77 | -92.96 |
| 6368 | 4539518 | Howe 2014 | 3708849455 | 43732113 | 41.77 | -92.96 |
| 6368 | 4539519 | Howe 2014 | 7602022910 | 94271386 | 41.77 | -92.96 |
| 6368 | 4539520 | Howe 2014 | 7772737156 | 95625033 | 41.77 | -92.96 |
| 6368 | 4539521 | Howe 2014 | 5639635894 | 70989994 | 41.77 | -92.96 |
| 6368 | 4539522 | Howe 2014 | 8298450011 | 105967276 | 41.77 | -92.96 |
| 6368 | 4539523 | Howe 2014 | 8223202056 | 104952205 | 41.77 | -92.96 |
| 6368 | 4539524 | Howe 2014 | 7940446217 | 100671525 | 41.77 | -92.96 |
| 6368 | 4539525 | Howe 2014 | 7820448206 | 100138529 | 41.77 | -92.96 |
| 6368 | 4539526 | Howe 2014 | 7868969289 | 100689719 | 41.77 | -92.96 |
| 6368 | 4539527 | Howe 2014 | 7664935098 | 98496588 | 41.77 | -92.96 |
| 6368 | 4539528 | Howe 2014 | 37968936507 | 520346510 | 41.77 | -92.96 |
| 6377 | 4539578 | Howe 2014 | 9746152379 | 137054866 | 41.83 | -93.01 |
| 6377 | 4539579 | Howe 2014 | 11583241547 | 167518968 | 41.83 | -93.01 |
| 6377 | 4539580 | Howe 2014 | 12351703559 | 179109050 | 41.83 | -93.01 |
| 6377 | 4539581 | Howe 2014 | 11004781140 | 158922931 | 41.83 | -93.01 |
| 6377 | 4539582 | Howe 2014 | 11307462276 | 167339991 | 41.83 | -93.01 |
| 6377 | 4539583 | Howe 2014 | 12018340835 | 176436132 | 41.83 | -93.01 |
| 6377 | 4539584 | Howe 2014 | 11999335511 | 175744326 | 41.83 | -93.01 |
| 6377 | 4539571 | Howe 2014 | 18328576290 | 213497494 | 41.83 | -93.01 |
| 6377 | 4539572 | Howe 2014 | 18724092302 | 217514697 | 41.83 | -93.01 |
| 6377 | 4539573 | Howe 2014 | 18569808348 | 217164987 | 41.83 | -93.01 |
| 6377 | 4539574 | Howe 2014 | 17649818427 | 206083276 | 41.83 | -93.01 |
| 6377 | 4539575 | Howe 2014 | 19954890565 | 233720367 | 41.83 | -93.01 |
| 6377 | 4539576 | Howe 2014 | 18582589285 | 216366290 | 41.83 | -93.01 |
| 6377 | 4539577 | Howe 2014 | 18579802988 | 215825187 | 41.83 | -93.01 |
| 6377 | 4539585 | Howe 2014 | 1621837703 | 25377110 | 41.83 | -93.01 |
| 6377 | 4539586 | Howe 2014 | 4512525899 | 64285342 | 41.83 | -93.01 |
| 6377 | 4539587 | Howe 2014 | 4452926593 | 65147859 | 41.83 | -93.01 |
| 6377 | 4539588 | Howe 2014 | 4546163216 | 66649310 | 41.83 | -93.01 |
| 6377 | 4539589 | Howe 2014 | 4589553132 | 66626658 | 41.83 | -93.01 |
| 6377 | 4539590 | Howe 2014 | 4582621593 | 66749607 | 41.83 | -93.01 |
| 6377 | 4539591 | Howe 2014 | 4565995156 | 65927593 | 41.83 | -93.01 |
| 6377 | 4539592 | Howe 2014 | 4586417918 | 49874608 | 41.83 | -93.01 |
| 6377 | 4539593 | Howe 2014 | 4175070745 | 49910017 | 41.83 | -93.01 |
| 6377 | 4539594 | Howe 2014 | 4216911842 | 49522529 | 41.83 | -93.01 |
| 13620 | 4633085 | Howe 2016 | 4805128530 | 47575530 | 41.92 | -93.75 |
| 13620 | 4633087 | Howe 2016 | 2527541766 | 25025166 | 41.92 | -93.75 |
| 13620 | 4633088 | Howe 2016 | 5290553720 | 52381720 | 41.92 | -93.75 |
| 13620 | 4633089 | Howe 2016 | 7376359058 | 73033258 | 41.92 | -93.75 |
| 13620 | 4633090 | Howe 2016 | 6535028250 | 64703250 | 41.92 | -93.75 |
| 13620 | 4633092 | Howe 2016 | 7611262838 | 75359038 | 41.92 | -93.75 |
| 13620 | 4633093 | Howe 2016 | 456193568 | 4516768 | 41.92 | -93.75 |
| 13620 | 4633098 | Howe 2016 | 5533218340 | 54784340 | 41.92 | -93.75 |
| 13620 | 4633188 | Howe 2016 | 4315928364 | 42731964 | 41.92 | -93.75 |
| 13620 | 4635362 | Howe 2016 | 20524028604 | 203208204 | 41.92 | -93.75 |
| 13620 | 4633120 | Howe 2016 | 7747986740 | 76712740 | 41.92 | -93.75 |
| 13620 | 4633122 | Howe 2016 | 8778023120 | 86911120 | 41.92 | -93.75 |
| 11953 | 4470255 | Hultman 2015 | 2143945902 | 18806543 | 64.68 | -157.83 |
| 11953 | 4470378 | Hultman 2015 | 4143700272 | 36348248 | 64.68 | -157.83 |
| 11953 | 4470253 | Hultman 2015 | 1712528748 | 15022182 | 64.68 | -157.83 |
| 11953 | 4470382 | Hultman 2015 | 4374118212 | 38369458 | 64.68 | -157.83 |
| 11953 | 4470254 | Hultman 2015 | 2160239580 | 18949470 | 64.68 | -157.83 |
| 11953 | 4470381 | Hultman 2015 | 4436027166 | 38912519 | 64.68 | -157.83 |
| 11953 | 4470315 | Hultman 2015 | 2508737466 | 22006469 | 64.68 | -157.83 |
| 11953 | 4469340 | Hultman 2015 | 8421304260 | 73871090 | 64.68 | -157.83 |
| 11953 | 4469540 | Hultman 2015 | 3542570292 | 31075178 | 64.68 | -157.83 |
| 11953 | 4469538 | Hultman 2015 | 3482173434 | 30545381 | 64.68 | -157.83 |
| 11953 | 4470010 | Hultman 2015 | 4664199306 | 40914029 | 64.68 | -157.83 |
| 11953 | 4470009 | Hultman 2015 | 4667382870 | 40941955 | 64.68 | -157.83 |
| 3321 | 4513030 | Kavamura 2018 | 125814492 | 1532644 | -9.07 | -40.33 |
| 3321 | 4513031 | Kavamura 2018 | 257135719 | 2382249 | -9.07 | -40.33 |
| 3321 | 4513032 | Kavamura 2018 | 257469392 | 2282295 | -9.07 | -40.33 |
| 3321 | 4513033 | Kavamura 2018 | 50636933 | 584923 | -9.07 | -40.33 |
| 3321 | 4513034 | Kavamura 2018 | 176569232 | 1708373 | -9.07 | -40.33 |
| 3321 | 4513035 | Kavamura 2018 | 225453657 | 2348555 | -9.07 | -40.33 |
| 17194 | 4689235 | Kerfahi 2018 | 555132152 | 3124430 | 78.54 | 11.44 |
| 17194 | 4689233 | Kerfahi 2018 | 561830320 | 3140836 | 78.54 | 11.44 |
| 17194 | 4689234 | Kerfahi 2018 | 501747813 | 2809121 | 78.54 | 11.44 |
| 17194 | 4689236 | Kerfahi 2018 | 592485956 | 3350243 | 78.54 | 11.44 |
| 17194 | 4689231 | Kerfahi 2018 | 325298665 | 1822285 | 78.54 | 11.44 |
| 17194 | 4689240 | Kerfahi 2018 | 112874758 | 636690 | 78.54 | 11.44 |
| 17194 | 4689232 | Kerfahi 2018 | 284350629 | 1595602 | 78.54 | 11.44 |
| 17194 | 4689230 | Kerfahi 2018 | 385189960 | 2163027 | 78.54 | 11.44 |
| 17194 | 4689239 | Kerfahi 2018 | 575792275 | 3178500 | 78.54 | 11.44 |
| 17194 | 4689238 | Kerfahi 2018 | 489611291 | 2586211 | 78.54 | 11.44 |
| 17194 | 4689228 | Kerfahi 2018 | 652155617 | 3552036 | 78.54 | 11.44 |
| 17194 | 4689237 | Kerfahi 2018 | 348854546 | 1912205 | 78.54 | 11.44 |
| 18555 | 4704031 | Kerfahi 2018 | 463935309 | 2503972 | 4.65 | 114.52 |
| 18555 | 4704028 | Kerfahi 2018 | 419434430 | 2262162 | 4.65 | 114.52 |
| 18555 | 4704036 | Kerfahi 2018 | 536504000 | 2918609 | 4.65 | 114.52 |
| 18555 | 4704040 | Kerfahi 2018 | 397057159 | 2140650 | 4.65 | 114.52 |
| 18555 | 4704026 | Kerfahi 2018 | 578855358 | 3148383 | 4.65 | 114.52 |
| 18555 | 4704033 | Kerfahi 2018 | 367849677 | 1981122 | 4.65 | 114.52 |
| 79383 | 4737786 | Kerfahi 2018 | 568646691 | 3144781 | 3.14 | 101.38 |
| 79383 | 4737789 | Kerfahi 2018 | 611946111 | 3400585 | 2.03 | 103.82 |
| 79383 | 4737787 | Kerfahi 2018 | 606969018 | 3371364 | 2.03 | 103.82 |
| 79383 | 4737783 | Kerfahi 2018 | 681249131 | 3774990 | 2.03 | 103.82 |
| 79383 | 4737793 | Kerfahi 2018 | 595748742 | 3306532 | 1.91 | 103.18 |
| 79383 | 4737790 | Kerfahi 2018 | 602951902 | 3356630 | 1.91 | 103.18 |
| 79383 | 4737784 | Kerfahi 2018 | 578138936 | 3163050 | 1.91 | 103.18 |
| 79383 | 4737782 | Kerfahi 2018 | 645292060 | 3612053 | 2.51 | 104.09 |
| 79383 | 4737792 | Kerfahi 2018 | 765707108 | 4432158 | 2.51 | 104.09 |
| 79383 | 4737785 | Kerfahi 2018 | 779404589 | 4379781 | 2.51 | 104.09 |
| 79383 | 4737777 | Kerfahi 2018 | 827601973 | 4800016 | 69.07 | -105.03 |
| 79383 | 4737788 | Kerfahi 2018 | 669114809 | 3724183 | 69.07 | -105.03 |
| 79383 | 4737781 | Kerfahi 2018 | 784985600 | 4786750 | 69.07 | -105.03 |
| 79383 | 4737791 | Kerfahi 2018 | 848750412 | 4906802 | 74.28 | -20.32 |
| 79383 | 4737779 | Kerfahi 2018 | 674748055 | 4094186 | 74.28 | -20.32 |
| 14949 | 4664136 | Kumaresan 2017 | 775779881 | 4191866 | -29.16 | 116.79 |
| 14949 | 4664135 | Kumaresan 2017 | 605024488 | 3274022 | -29.16 | 116.79 |
| 14949 | 4664124 | Kumaresan 2017 | 564566045 | 3027365 | -29.16 | 116.79 |
| 14949 | 4664125 | Kumaresan 2017 | 990862622 | 5356084 | -29.16 | 116.79 |
| 14949 | 4664131 | Kumaresan 2017 | 548206461 | 2971565 | -29.16 | 116.79 |
| 14949 | 4664147 | Kumaresan 2017 | 409821189 | 2185128 | -29.16 | 116.79 |
| 14949 | 4664132 | Kumaresan 2017 | 743136365 | 4092658 | -29.16 | 116.79 |
| 14949 | 4664134 | Kumaresan 2017 | 891843317 | 4879625 | -29.16 | 116.79 |
| 14949 | 4664137 | Kumaresan 2017 | 637309471 | 3457837 | -29.16 | 116.79 |
| 14949 | 4664129 | Kumaresan 2017 | 878981737 | 4847947 | -29.16 | 116.79 |
| 14949 | 4664127 | Kumaresan 2017 | 899265650 | 4949673 | -29.16 | 116.79 |
| 14949 | 4664128 | Kumaresan 2017 | 774612671 | 4247839 | -29.16 | 116.79 |
| 14949 | 4664138 | Kumaresan 2017 | 540034515 | 2912064 | -29.16 | 116.79 |
| 14949 | 4664133 | Kumaresan 2017 | 757840706 | 4160749 | -29.16 | 116.79 |
| 14949 | 4664130 | Kumaresan 2017 | 870106508 | 4700891 | -29.16 | 116.79 |
| 14949 | 4664126 | Kumaresan 2017 | 701416826 | 3788763 | -29.16 | 116.79 |
| 8742 | 4565459 | Luis_Castañeda 2017 | 61669011 | 141694 | -33.58 | -71.37 |
| 8742 | 4565463 | Luis_Castañeda 2017 | 78236955 | 178272 | -32.87 | -71.12 |
| 8742 | 4565458 | Luis_Castañeda 2017 | 87262168 | 195138 | -34.60 | -71.12 |
| 8742 | 4565460 | Luis_Castañeda 2017 | 84497008 | 189372 | -33.58 | -71.37 |
| 8742 | 4565462 | Luis_Castañeda 2017 | 94090245 | 208095 | -32.87 | -71.12 |
| 8742 | 4565461 | Luis_Castañeda 2017 | 86785396 | 201266 | -34.60 | -71.12 |
| 7774 | 4478038 | Mendes 2014 | 73786733 | 140141 | -11.74 | -56.25 |
| 7774 | 4478222 | Mendes 2014 | 74737637 | 142020 | -11.74 | -56.25 |
| 7774 | 4478283 | Mendes 2014 | 73942895 | 140261 | -11.74 | -56.25 |
| 7774 | 4478290 | Mendes 2014 | 77192998 | 147028 | -11.68 | -55.84 |
| 7774 | 4478292 | Mendes 2014 | 68378389 | 129829 | -11.68 | -55.84 |
| 7774 | 4478291 | Mendes 2014 | 59112731 | 112056 | -11.68 | -55.84 |
| 7774 | 4478294 | Mendes 2014 | 72823122 | 138556 | -11.68 | -55.84 |
| 7774 | 4478936 | Mendes 2014 | 72235815 | 137939 | -11.68 | -55.84 |
| 7774 | 4478937 | Mendes 2014 | 76798363 | 146385 | -11.68 | -55.84 |
| 7774 | 4478938 | Mendes 2014 | 66071784 | 126119 | -11.68 | -55.84 |
| 7774 | 4477751 | Mendes 2014 | 74088056 | 141552 | -11.68 | -55.84 |
| 7774 | 4478939 | Mendes 2014 | 69961802 | 133146 | -11.74 | -56.25 |
| 7774 | 4479311 | Mendes 2014 | 79557283 | 151804 | -11.74 | -56.25 |
| 7774 | 4478940 | Mendes 2014 | 72236578 | 138073 | -11.74 | -56.25 |
| 7774 | 4478941 | Mendes 2014 | 75538772 | 143858 | -11.74 | -56.25 |
| 7774 | 4478943 | Mendes 2014 | 64862607 | 123137 | -11.74 | -56.25 |
| 7774 | 4477749 | Mendes 2014 | 78885433 | 151054 | -11.68 | -55.84 |
| 7774 | 4477755 | Mendes 2014 | 73154894 | 140546 | -11.68 | -55.84 |
| 7774 | 4477757 | Mendes 2014 | 75485385 | 144908 | -11.68 | -55.84 |
| 7774 | 4477789 | Mendes 2014 | 71757836 | 137109 | -11.68 | -55.84 |
| 7774 | 4477790 | Mendes 2014 | 75459983 | 144587 | -11.68 | -55.84 |
| 7774 | 4478030 | Mendes 2014 | 75354498 | 145214 | -11.68 | -55.84 |
| 7774 | 4478037 | Mendes 2014 | 65489369 | 124076 | -11.74 | -56.25 |
| 7774 | 4478934 | Mendes 2014 | 103182378 | 113953 | -11.74 | -56.25 |
| 1519 | 4497370 | Mendes 2015 | 1452524300 | 15521548 | -11.68 | -55.84 |
| 1519 | 4497371 | Mendes 2015 | 1585023687 | 16901918 | -11.68 | -55.84 |
| 1519 | 4497372 | Mendes 2015 | 1641076813 | 17445400 | -11.71 | -55.79 |
| 1519 | 4497373 | Mendes 2015 | 1958519064 | 20851107 | -11.71 | -55.79 |
| 1519 | 4497374 | Mendes 2015 | 1440853866 | 15240438 | -11.71 | -55.79 |
| 1519 | 4497375 | Mendes 2015 | 1938015010 | 20635352 | -11.71 | -55.79 |
| 1519 | 4497376 | Mendes 2015 | 1420896267 | 15155481 | -11.71 | -55.79 |
| 1519 | 4497377 | Mendes 2015 | 1994863535 | 21256135 | -11.71 | -55.79 |
| 1519 | 4497378 | Mendes 2015 | 1305482377 | 13926576 | -11.71 | -55.79 |
| 1519 | 4497379 | Mendes 2015 | 2281777227 | 24375188 | -11.74 | -56.26 |
| 1519 | 4497380 | Mendes 2015 | 1529424461 | 16303010 | -11.74 | -56.26 |
| 1519 | 4497381 | Mendes 2015 | 1842131597 | 19634073 | -11.74 | -56.26 |
| 1519 | 4497382 | Mendes 2015 | 1623856791 | 17298270 | -11.74 | -56.26 |
| 1519 | 4497383 | Mendes 2015 | 1592168131 | 16954685 | -11.74 | -56.26 |
| 1519 | 4497384 | Mendes 2015 | 1603768269 | 17133206 | -11.74 | -56.26 |
| 1519 | 4497385 | Mendes 2015 | 1833433977 | 19529714 | -11.74 | -56.26 |
| 1519 | 4497386 | Mendes 2015 | 1439335287 | 15342158 | -11.74 | -56.26 |
| 1519 | 4497387 | Mendes 2015 | 1762026012 | 18901443 | -11.74 | -56.25 |
| 1519 | 4497388 | Mendes 2015 | 1338341598 | 14368635 | -11.74 | -56.25 |
| 1519 | 4497389 | Mendes 2015 | 1535671061 | 16373863 | -11.68 | -55.84 |
| 1519 | 4497390 | Mendes 2015 | 1917707251 | 20423704 | -11.68 | -55.84 |
| 1519 | 4497391 | Mendes 2015 | 2339288507 | 25021054 | -11.74 | -56.25 |
| 1519 | 4497392 | Mendes 2015 | 1253546383 | 13402895 | -11.74 | -56.25 |
| 1519 | 4497393 | Mendes 2015 | 1841976159 | 19818763 | -11.74 | -56.25 |
| 1519 | 4497394 | Mendes 2015 | 1494681282 | 15951446 | -11.74 | -56.25 |
| 1519 | 4497395 | Mendes 2015 | 1442975076 | 15428263 | -11.74 | -56.25 |
| 1519 | 4497396 | Mendes 2015 | 2759962619 | 29499368 | -11.74 | -56.25 |
| 1519 | 4497397 | Mendes 2015 | 1717616735 | 18337617 | -11.68 | -55.84 |
| 1519 | 4497398 | Mendes 2015 | 1368742061 | 14554642 | -11.68 | -55.84 |
| 1519 | 4497399 | Mendes 2015 | 2091680663 | 22245990 | -11.68 | -55.84 |
| 1519 | 4497400 | Mendes 2015 | 1652011068 | 17618750 | -11.68 | -55.84 |
| 1519 | 4497401 | Mendes 2015 | 1232841286 | 13127073 | -11.68 | -55.84 |
| 1519 | 4497402 | Mendes 2015 | 1567594935 | 16687613 | -11.68 | -55.84 |
| 1519 | 4497403 | Mendes 2015 | 980702897 | 10477278 | -11.68 | -55.01 |
| 1519 | 4497404 | Mendes 2015 | 1661893955 | 17817773 | -11.68 | -55.01 |
| 1519 | 4497405 | Mendes 2015 | 1349881717 | 14427611 | -11.68 | -55.01 |
| 1519 | 4497406 | Mendes 2015 | 1835174499 | 19593526 | -11.68 | -55.01 |
| 1519 | 4497407 | Mendes 2015 | 1691682287 | 18119871 | -11.68 | -55.84 |
| 1519 | 4497408 | Mendes 2015 | 2985513423 | 31966967 | -11.68 | -55.84 |
| 1519 | 4497409 | Mendes 2015 | 1449085730 | 15455238 | -11.68 | -55.84 |
| 1519 | 4497410 | Mendes 2015 | 2340921723 | 25017093 | -11.68 | -55.84 |
| 1519 | 4497411 | Mendes 2015 | 1297860799 | 13908400 | -11.68 | -55.84 |
| 1519 | 4497412 | Mendes 2015 | 2224899417 | 23867305 | -11.68 | -55.84 |
| 4362 | 4521497 | Mendes 2015 | 21258091 | 116867 | -25.07 | -47.92 |
| 4362 | 4521499 | Mendes 2015 | 129226387 | 544766 | -25.09 | -47.96 |
| 4362 | 4521501 | Mendes 2015 | 168587759 | 686124 | -25.07 | -47.92 |
| 4362 | 4521503 | Mendes 2015 | 13524850 | 72900 | -25.07 | -47.92 |
| 4362 | 4521505 | Mendes 2015 | 228098028 | 939772 | -25.09 | -47.96 |
| 4362 | 4521507 | Mendes 2015 | 115636155 | 481069 | -25.07 | -47.92 |
| 4362 | 4521509 | Mendes 2015 | 23893592 | 131160 | -25.07 | -47.92 |
| 4362 | 4521511 | Mendes 2015 | 282848961 | 1172726 | -25.09 | -47.96 |
| 12156 | 4612989 | Mendes 2017 | 323757692 | 1453973 | -3.27 | -60.20 |
| 12156 | 4612990 | Mendes 2017 | 482515714 | 1993524 | -3.27 | -60.20 |
| 12156 | 4612991 | Mendes 2017 | 288274591 | 1293288 | -3.27 | -60.20 |
| 12156 | 4612674 | Mendes 2017 | 126509036 | 648283 | -3.27 | -60.20 |
| 12156 | 4612902 | Mendes 2017 | 189370923 | 822058 | -3.27 | -60.20 |
| 12156 | 4612985 | Mendes 2017 | 226005595 | 1124037 | -3.27 | -60.20 |
| 12156 | 4612986 | Mendes 2017 | 95724666 | 485804 | -3.27 | -60.20 |
| 12156 | 4612675 | Mendes 2017 | 252356997 | 1093748 | -3.27 | -60.20 |
| 12156 | 4612676 | Mendes 2017 | 55537782 | 290957 | -3.27 | -60.20 |
| 12156 | 4612677 | Mendes 2017 | 195881569 | 953167 | -3.27 | -60.20 |
| 12156 | 4612679 | Mendes 2017 | 217088770 | 1035679 | -3.27 | -60.20 |
| 12156 | 4612898 | Mendes 2017 | 83017588 | 398264 | -3.27 | -60.20 |
| 12156 | 4612899 | Mendes 2017 | 222051443 | 1033077 | -3.27 | -60.20 |
| 12156 | 4612900 | Mendes 2017 | 96905238 | 487960 | -3.27 | -60.20 |
| 12156 | 4612901 | Mendes 2017 | 87275720 | 418750 | -3.27 | -60.20 |
| 12156 | 4615280 | Mendes 2017 | 86584758 | 462103 | -22.67 | -48.17 |
| 12156 | 4615281 | Mendes 2017 | 119318640 | 601490 | -22.67 | -48.17 |
| 12156 | 4615282 | Mendes 2017 | 158438846 | 698886 | -22.67 | -48.17 |
| 12156 | 4615218 | Mendes 2017 | 224623137 | 994647 | -22.67 | -48.17 |
| 12156 | 4615275 | Mendes 2017 | 210639683 | 943704 | -22.67 | -48.17 |
| 12156 | 4615276 | Mendes 2017 | 178133705 | 793923 | -22.67 | -48.17 |
| 12156 | 4615277 | Mendes 2017 | 152045857 | 719426 | -22.67 | -48.17 |
| 12156 | 4615220 | Mendes 2017 | 184799646 | 884816 | -22.67 | -48.17 |
| 12156 | 4615241 | Mendes 2017 | 241078476 | 1065596 | -22.67 | -48.17 |
| 12156 | 4615243 | Mendes 2017 | 212087753 | 968822 | -22.67 | -48.17 |
| 12156 | 4615217 | Mendes 2017 | 168424322 | 784516 | -22.67 | -48.17 |
| 12156 | 4620456 | Mendes 2017 | 81018867 | 394894 | -22.67 | -48.17 |
| 12156 | 4615270 | Mendes 2017 | 250128856 | 1083940 | -22.67 | -48.17 |
| 12156 | 4615272 | Mendes 2017 | 249058646 | 1080277 | -22.67 | -48.17 |
| 12156 | 4615273 | Mendes 2017 | 174124328 | 792431 | -22.67 | -48.17 |
| 12156 | 4615274 | Mendes 2017 | 247609161 | 1062667 | -22.67 | -48.17 |
| 12953 | 4622702 | Meneghine 2017 | 2969248149 | 31764931 | -23.58 | -47.59 |
| 12953 | 4622703 | Meneghine 2017 | 4163450321 | 44539294 | -23.58 | -47.59 |
| 12953 | 4622704 | Meneghine 2017 | 2161027347 | 23164262 | -23.58 | -47.59 |
| 3731 | 4530393 | Meyer 2017 | 16335764619 | 98185139 | -10.17 | -62.82 |
| 3731 | 4535541 | Meyer 2017 | 16184171205 | 96165003 | -10.17 | -62.82 |
| 3731 | 4535545 | Meyer 2017 | 16499910405 | 100776121 | -10.17 | -62.82 |
| 3731 | 4535560 | Meyer 2017 | 16468726157 | 100377612 | -10.17 | -62.82 |
| 3731 | 4535559 | Meyer 2017 | 15790025427 | 87821959 | -10.17 | -62.82 |
| 3731 | 4536768 | Meyer 2017 | 14685329089 | 71157224 | -10.17 | -62.82 |
| 3731 | 4535539 | Meyer 2017 | 16938597086 | 105134940 | -10.17 | -62.82 |
| 3731 | 4535553 | Meyer 2017 | 16719488533 | 102022509 | -10.17 | -62.82 |
| 3731 | 4546395 | Meyer 2017 | 17999861638 | 119986013 | -10.17 | -62.82 |
| 3731 | 4536139 | Meyer 2017 | 17631239942 | 114603805 | -10.17 | -62.82 |
| 3731 | 4546394 | Meyer 2017 | 17058292904 | 105950943 | -10.17 | -62.82 |
| 3731 | 4537210 | Meyer 2017 | 14733358729 | 79572931 | -10.17 | -62.82 |
| 3731 | 4536464 | Meyer 2017 | 14779368877 | 80224980 | -10.17 | -62.82 |
| 3731 | 4546658 | Meyer 2017 | 16712984445 | 101741019 | -10.17 | -62.82 |
| 3731 | 4536477 | Meyer 2017 | 16793645730 | 102976326 | -10.17 | -62.82 |
| 3731 | 4537330 | Meyer 2017 | 16849928413 | 104390923 | -10.17 | -62.82 |
| 3731 | 4545668 | Meyer 2017 | 17090468370 | 107708580 | -10.17 | -62.82 |
| 3731 | 4537326 | Meyer 2017 | 16540649794 | 101445480 | -10.17 | -62.82 |
| 3731 | 4537328 | Meyer 2017 | 17000473297 | 107469820 | -10.17 | -62.82 |
| 3731 | 4537324 | Meyer 2017 | 16099183251 | 89884388 | -10.17 | -62.82 |
| 3731 | 4536577 | Meyer 2017 | 16032528789 | 88815333 | -10.17 | -62.82 |
| 11261 | 4597439 | Miura 2016 | 145695467 | 713843 | -4.56 | 105.41 |
| 11261 | 4597448 | Miura 2016 | 153583796 | 795066 | -4.56 | 105.41 |
| 11261 | 4597438 | Miura 2016 | 262122963 | 1270366 | -4.56 | 105.41 |
| 11261 | 4597449 | Miura 2016 | 259021467 | 1405455 | -4.56 | 105.41 |
| 11261 | 4597441 | Miura 2016 | 102507103 | 564351 | -4.56 | 105.41 |
| 11261 | 4597445 | Miura 2016 | 235654305 | 1148813 | -4.56 | 105.41 |
| 11261 | 4597446 | Miura 2016 | 206972775 | 1050789 | -4.56 | 105.41 |
| 11261 | 4597437 | Miura 2016 | 184262421 | 999010 | -4.56 | 105.41 |
| 11261 | 4597447 | Miura 2016 | 200269756 | 1123082 | -4.56 | 105.41 |
| 11261 | 4597435 | Miura 2016 | 294060502 | 1404353 | -4.56 | 105.41 |
| 11261 | 4597443 | Miura 2016 | 130906936 | 652648 | -4.56 | 105.41 |
| 11261 | 4597434 | Miura 2016 | 172252285 | 898866 | -4.56 | 105.41 |
| 11261 | 4597436 | Miura 2016 | 196889323 | 989291 | -4.56 | 105.41 |
| 11261 | 4597440 | Miura 2016 | 327237058 | 1567417 | -4.56 | 105.41 |
| 11261 | 4597444 | Miura 2016 | 214821518 | 1045866 | -4.56 | 105.41 |
| 11261 | 4597442 | Miura 2016 | 233895479 | 1126156 | -4.56 | 105.41 |
| 10854 | 4582104 | Navarrete 2015 | 49094133 | 196990 | -22.41 | -47.38 |
| 10854 | 4582105 | Navarrete 2015 | 43089324 | 173507 | -22.41 | -47.38 |
| 10854 | 4582106 | Navarrete 2015 | 63759972 | 252223 | -22.41 | -47.38 |
| 10854 | 4582107 | Navarrete 2015 | 156821373 | 601075 | -22.41 | -47.38 |
| 10854 | 4582108 | Navarrete 2015 | 214844156 | 808603 | -22.41 | -47.38 |
| 10854 | 4582109 | Navarrete 2015 | 89892936 | 343369 | -22.41 | -47.38 |
| 10854 | 4582110 | Navarrete 2015 | 130888890 | 489532 | -22.41 | -47.38 |
| 10854 | 4582111 | Navarrete 2015 | 86067528 | 339951 | -22.41 | -47.38 |
| 10854 | 4582112 | Navarrete 2015 | 92769021 | 350530 | -22.41 | -47.38 |
| 10854 | 4582113 | Navarrete 2015 | 79998187 | 295302 | -22.41 | -47.38 |
| 10854 | 4582114 | Navarrete 2015 | 52357824 | 194678 | -22.41 | -47.38 |
| 10854 | 4582115 | Navarrete 2015 | 35201469 | 138190 | -22.41 | -47.38 |
| 10854 | 4582116 | Navarrete 2015 | 205150663 | 912493 | -22.41 | -47.38 |
| 10854 | 4582117 | Navarrete 2015 | 122410720 | 554451 | -22.41 | -47.38 |
| 10854 | 4582118 | Navarrete 2015 | 96898703 | 447980 | -22.41 | -47.38 |
| 10854 | 4582119 | Navarrete 2015 | 128564285 | 537684 | -22.41 | -47.38 |
| 10854 | 4582120 | Navarrete 2015 | 41812511 | 163018 | -22.41 | -47.38 |
| 10854 | 4582121 | Navarrete 2015 | 35630906 | 137828 | -22.41 | -47.38 |
| 10854 | 4582122 | Navarrete 2015 | 37517394 | 150725 | -22.41 | -47.38 |
| 10854 | 4582123 | Navarrete 2015 | 97546246 | 372361 | -22.41 | -47.38 |
| 10854 | 4582124 | Navarrete 2015 | 126768250 | 480630 | -22.41 | -47.38 |
| 10854 | 4582125 | Navarrete 2015 | 157582145 | 586856 | -22.41 | -47.38 |
| 10854 | 4582126 | Navarrete 2015 | 157537137 | 619326 | -22.41 | -47.38 |
| 10854 | 4582127 | Navarrete 2015 | 91391385 | 349119 | -22.41 | -47.38 |
| 10854 | 4582128 | Navarrete 2015 | 198242156 | 790759 | -22.41 | -47.38 |
| 10854 | 4582129 | Navarrete 2015 | 119230868 | 445603 | -22.41 | -47.38 |
| 10854 | 4582130 | Navarrete 2015 | 91290010 | 333588 | -22.41 | -47.38 |
| 10854 | 4582131 | Navarrete 2015 | 69071883 | 249792 | -22.41 | -47.38 |
| 10854 | 4582132 | Navarrete 2015 | 117971632 | 451569 | -22.41 | -47.38 |
| 10854 | 4582133 | Navarrete 2015 | 96385109 | 379678 | -22.41 | -47.38 |
| 10854 | 4582134 | Navarrete 2015 | 145809468 | 558384 | -22.41 | -47.38 |
| 10854 | 4582135 | Navarrete 2015 | 75193927 | 278937 | -22.41 | -47.38 |
| 10854 | 4582136 | Navarrete 2015 | 39948409 | 153081 | -22.41 | -47.38 |
| 10854 | 4582137 | Navarrete 2015 | 50341767 | 188924 | -22.41 | -47.38 |
| 10854 | 4582138 | Navarrete 2015 | 71731090 | 268275 | -22.41 | -47.38 |
| 10854 | 4582139 | Navarrete 2015 | 48235670 | 182497 | -22.41 | -47.38 |
| 10854 | 4582140 | Navarrete 2015 | 69532834 | 261086 | -22.41 | -47.38 |
| 10854 | 4582141 | Navarrete 2015 | 63644298 | 243083 | -22.41 | -47.38 |
| 10854 | 4582142 | Navarrete 2015 | 146135429 | 552597 | -22.41 | -47.38 |
| 10854 | 4582143 | Navarrete 2015 | 110697412 | 443764 | -22.41 | -47.38 |
| 10854 | 4582144 | Navarrete 2015 | 151235854 | 563293 | -22.41 | -47.38 |
| 10854 | 4582145 | Navarrete 2015 | 91592446 | 348374 | -22.41 | -47.38 |
| 10854 | 4582146 | Navarrete 2015 | 229026435 | 872055 | -22.41 | -47.38 |
| 10854 | 4582147 | Navarrete 2015 | 167899889 | 701215 | -22.41 | -47.38 |
| 10854 | 4582148 | Navarrete 2015 | 42832283 | 177380 | -22.41 | -47.38 |
| 10854 | 4582149 | Navarrete 2015 | 237443487 | 882557 | -22.41 | -47.38 |
| 10854 | 4582150 | Navarrete 2015 | 46609894 | 179119 | -22.41 | -47.38 |
| 10854 | 4582151 | Navarrete 2015 | 112772969 | 447748 | -22.41 | -47.38 |
| 10854 | 4582152 | Navarrete 2015 | 153403936 | 579705 | -22.41 | -47.38 |
| 10854 | 4582153 | Navarrete 2015 | 180335902 | 741799 | -22.41 | -47.38 |
| 10854 | 4582154 | Navarrete 2015 | 124467080 | 497497 | -22.41 | -47.38 |
| 10854 | 4582155 | Navarrete 2015 | 84225041 | 337758 | -22.41 | -47.38 |
| 10854 | 4582156 | Navarrete 2015 | 55769135 | 221454 | -22.41 | -47.38 |
| 10854 | 4582157 | Navarrete 2015 | 93737989 | 364943 | -22.41 | -47.38 |
| 10854 | 4582158 | Navarrete 2015 | 192940499 | 747782 | -22.41 | -47.38 |
| 10854 | 4582159 | Navarrete 2015 | 79707501 | 308912 | -22.41 | -47.38 |
| 10854 | 4582160 | Navarrete 2015 | 72816398 | 293141 | -22.41 | -47.38 |
| 837 | 4493650 | Navarrete 2015 | 2253115473 | 23319588 | -14.33 | -54.37 |
| 837 | 4493651 | Navarrete 2015 | 984956429 | 10101224 | -14.33 | -54.37 |
| 837 | 4493652 | Navarrete 2015 | 2297811383 | 23648292 | -14.33 | -54.37 |
| 837 | 4493653 | Navarrete 2015 | 3602706792 | 37017180 | -13.38 | -54.94 |
| 837 | 4493654 | Navarrete 2015 | 2209118929 | 22858762 | -13.38 | -54.94 |
| 837 | 4493655 | Navarrete 2015 | 2997747324 | 30848080 | -13.38 | -54.94 |
| 837 | 4493649 | Navarrete 2015 | 2536664461 | 26022580 | -15.20 | -54.06 |
| 837 | 4493544 | Navarrete 2015 | 2725848472 | 27945536 | -15.20 | -54.06 |
| 837 | 4493545 | Navarrete 2015 | 2772651355 | 28432282 | -15.20 | -54.06 |
| 837 | 4493892 | Navarrete 2015 | 7041966249 | 72049364 | -14.36 | -54.36 |
| 837 | 4493546 | Navarrete 2015 | 1033654661 | 11083752 | -14.36 | -54.36 |
| 837 | 4493893 | Navarrete 2015 | 5980079018 | 61788986 | -14.36 | -54.36 |
| 837 | 4493547 | Navarrete 2015 | 2321564826 | 23872968 | -13.37 | -54.91 |
| 837 | 4493548 | Navarrete 2015 | 2780516789 | 30062278 | -13.37 | -54.91 |
| 837 | 4493549 | Navarrete 2015 | 2735803610 | 27957742 | -13.37 | -54.91 |
| 13248 | 4626743 | Nguyen 2016 | 4403800990 | 43601990 | 21.09 | 105.73 |
| 13248 | 4626753 | Nguyen 2016 | 1218720433 | 4094343 | 21.09 | 105.73 |
| 13248 | 4626745 | Nguyen 2016 | 5390462819 | 53370919 | 21.09 | 105.73 |
| 13248 | 4626754 | Nguyen 2016 | 1219671314 | 4094343 | 21.09 | 105.73 |
| 13248 | 4626747 | Nguyen 2016 | 4433473780 | 43895780 | 21.09 | 105.73 |
| 13248 | 4626755 | Nguyen 2016 | 1381298306 | 4665752 | 21.09 | 105.73 |
| 13248 | 4626756 | Nguyen 2016 | 1382587302 | 4665752 | 21.09 | 105.73 |
| 1008 | 4485389 | Pan 2014 | 40423549 | 105010 | 51.97 | 5.64 |
| 1008 | 4485390 | Pan 2014 | 44905782 | 114709 | 51.97 | 5.64 |
| 1008 | 4485403 | Pan 2014 | 49728531 | 126331 | 51.97 | 5.64 |
| 1008 | 4485400 | Pan 2014 | 51665672 | 130016 | 51.97 | 5.64 |
| 1008 | 4485401 | Pan 2014 | 52226427 | 133309 | 51.97 | 5.64 |
| 1008 | 4485402 | Pan 2014 | 43656772 | 113565 | 51.97 | 5.64 |
| 1008 | 4485391 | Pan 2014 | 52721323 | 134389 | 51.97 | 5.64 |
| 1008 | 4485392 | Pan 2014 | 42073456 | 113883 | 51.97 | 5.64 |
| 1008 | 4485393 | Pan 2014 | 38425397 | 96793 | 51.97 | 5.64 |
| 1008 | 4485394 | Pan 2014 | 33521299 | 84053 | 51.97 | 5.64 |
| 1008 | 4485395 | Pan 2014 | 29433082 | 76677 | 51.97 | 5.64 |
| 1008 | 4485396 | Pan 2014 | 32356773 | 83399 | 51.97 | 5.64 |
| 1008 | 4485397 | Pan 2014 | 28573338 | 76859 | 51.97 | 5.64 |
| 1008 | 4485398 | Pan 2014 | 28396437 | 78248 | 51.97 | 5.64 |
| 1008 | 4485399 | Pan 2014 | 34738730 | 90340 | 51.97 | 5.64 |
| 3513 | 4514245 | Pandit 2015 | 387783372 | 2423783 | 23.81 | 70.98 |
| 3513 | 4514299 | Pandit 2015 | 322114449 | 2420832 | 23.79 | 71.01 |
| 3513 | 4543019 | Pandit 2015 | 282578916 | 2016127 | 23.03 | 70.22 |
| 3513 | 4543020 | Pandit 2015 | 467782469 | 3100617 | 23.91 | 70.54 |
| 3513 | 4543021 | Pandit 2015 | 235993491 | 2349820 | 23.94 | 70.19 |
| 3513 | 4543022 | Pandit 2015 | 343077214 | 3041048 | 23.83 | 69.52 |
| 3513 | 4543023 | Pandit 2015 | 308124473 | 2048827 | 23.94 | 70.19 |
| 271 | 4450130 | Ridl 2016 | 297444144 | 727529 | 48.99 | 14.16 |
| 271 | 4450131 | Ridl 2016 | 276871390 | 665714 | 48.99 | 14.16 |
| 271 | 4450171 | Ridl 2016 | 286480433 | 712104 | 48.99 | 14.16 |
| 271 | 4450172 | Ridl 2016 | 308435859 | 756433 | 48.99 | 14.16 |
| 271 | 4450628 | Ridl 2016 | 245662269 | 584208 | 48.99 | 14.16 |
| 271 | 4450629 | Ridl 2016 | 275891786 | 655217 | 48.99 | 14.16 |
| 271 | 4450630 | Ridl 2016 | 325348558 | 794668 | 48.99 | 14.16 |
| 271 | 4450631 | Ridl 2016 | 301519047 | 735196 | 48.99 | 14.16 |
| 7877 | 4552602 | Smith 2016 | 1343608914 | 13294138 | 39.46 | -84.95 |
| 7877 | 4552603 | Smith 2016 | 1754754798 | 17361712 | 39.46 | -84.94 |
| 7877 | 4552621 | Smith 2016 | 2317770032 | 22931228 | 39.46 | -84.95 |
| 7877 | 4552622 | Smith 2016 | 1888167542 | 18668477 | 39.46 | -84.94 |
| 7877 | 4552607 | Smith 2016 | 2937315025 | 29058789 | 39.77 | -84.85 |
| 7877 | 4552597 | Smith 2016 | 1431745529 | 14169028 | 39.95 | -84.81 |
| 7877 | 4552598 | Smith 2016 | 1235728039 | 12229474 | 39.93 | -84.76 |
| 7877 | 4552599 | Smith 2016 | 2976499049 | 29455785 | 39.93 | -84.77 |
| 7877 | 4552600 | Smith 2016 | 2918474743 | 28877883 | 39.81 | -84.91 |
| 7877 | 4552601 | Smith 2016 | 1596035427 | 15793481 | 39.80 | -84.92 |
| 7877 | 4552604 | Smith 2016 | 1036580474 | 10258094 | 39.88 | -84.98 |
| 7877 | 4552605 | Smith 2016 | 4454201 | 44065 | 39.88 | -84.98 |
| 7877 | 4552606 | Smith 2016 | 2921679340 | 28897599 | 39.88 | -84.98 |
| 7877 | 4552609 | Smith 2016 | 2647555981 | 26201119 | 39.77 | -84.82 |
| 7877 | 4552608 | Smith 2016 | 1286824690 | 12735016 | 39.88 | -84.98 |
| 7877 | 4552610 | Smith 2016 | 2452638515 | 24272188 | 39.77 | -84.83 |
| 7877 | 4552611 | Smith 2016 | 1432525102 | 14174970 | 39.77 | -84.83 |
| 7877 | 4552612 | Smith 2016 | 1186464826 | 11739217 | 39.90 | -85.00 |
| 7877 | 4552613 | Smith 2016 | 3171919697 | 31391317 | 39.90 | -85.01 |
| 7877 | 4552614 | Smith 2016 | 1627589348 | 16105725 | 39.89 | -84.98 |
| 7877 | 4552615 | Smith 2016 | 2230030788 | 22069730 | 39.89 | -84.98 |
| 7877 | 4552616 | Smith 2016 | 1475556590 | 14599171 | 39.95 | -84.81 |
| 7877 | 4552626 | Smith 2016 | 3813242064 | 37717342 | 39.77 | -84.85 |
| 7877 | 4552617 | Smith 2016 | 1144230699 | 11311337 | 39.93 | -84.76 |
| 7877 | 4552618 | Smith 2016 | 1756407594 | 17335758 | 39.93 | -84.77 |
| 7877 | 4552619 | Smith 2016 | 1598448932 | 15798980 | 39.81 | -84.91 |
| 7877 | 4552620 | Smith 2016 | 1787588588 | 17680870 | 39.80 | -84.92 |
| 7877 | 4552623 | Smith 2016 | 1891570794 | 18674403 | 39.88 | -84.98 |
| 7877 | 4552624 | Smith 2016 | 3617528765 | 35762397 | 39.88 | -84.98 |
| 7877 | 4552625 | Smith 2016 | 1838277364 | 18164258 | 39.88 | -84.98 |
| 7877 | 4552630 | Smith 2016 | 3644801503 | 36009146 | 39.77 | -84.82 |
| 7877 | 4552627 | Smith 2016 | 3363860585 | 33236811 | 39.88 | -84.98 |
| 7877 | 4552628 | Smith 2016 | 4168197732 | 41202077 | 39.75 | -84.92 |
| 7877 | 4552629 | Smith 2016 | 2556441674 | 25292342 | 39.80 | -84.92 |
| 7877 | 4552631 | Smith 2016 | 1551039149 | 15338248 | 39.77 | -84.83 |
| 7877 | 4552632 | Smith 2016 | 1533060133 | 15163928 | 39.90 | -85.00 |
| 7877 | 4552633 | Smith 2016 | 1437615180 | 14220298 | 39.90 | -85.01 |
| 7877 | 4552634 | Smith 2016 | 1270377420 | 12563469 | 39.89 | -84.98 |
| 7877 | 4552635 | Smith 2016 | 1350535185 | 13345371 | 39.89 | -84.98 |
| 7877 | 4552636 | Smith 2016 | 1650655226 | 16318159 | 39.95 | -84.81 |
| 20476 | 4722923 | Song 2019 | 343116440 | 1893020 | 29.92 | 91.02 |
| 20476 | 4722924 | Song 2019 | 266192110 | 1459937 | 29.92 | 91.02 |
| 20476 | 4722925 | Song 2019 | 306101547 | 1660018 | 29.92 | 91.02 |
| 20476 | 4722926 | Song 2019 | 351578307 | 1896607 | 29.92 | 91.02 |
| 20476 | 4722927 | Song 2019 | 377869140 | 2052388 | 29.92 | 91.02 |
| 20476 | 4722928 | Song 2019 | 283730236 | 1539338 | 29.92 | 91.02 |
| 20476 | 4722929 | Song 2019 | 327324436 | 1788524 | 29.92 | 91.02 |
| 20476 | 4722930 | Song 2019 | 289141689 | 1571359 | 29.92 | 91.02 |
| 20476 | 4722931 | Song 2019 | 400355471 | 2165277 | 29.92 | 91.02 |
| 20476 | 4722932 | Song 2019 | 363881024 | 1978367 | 29.92 | 91.02 |
| 20476 | 4722933 | Song 2019 | 412437742 | 2245068 | 29.92 | 91.02 |
| 20476 | 4722934 | Song 2019 | 396603311 | 2148907 | 29.92 | 91.02 |
| 20476 | 4722935 | Song 2019 | 381461821 | 2072766 | 29.92 | 91.02 |
| 20476 | 4722936 | Song 2019 | 427680935 | 2302633 | 29.92 | 91.02 |
| 20476 | 4722937 | Song 2019 | 314579529 | 1704250 | 29.92 | 91.02 |
| 20476 | 4722938 | Song 2019 | 244093502 | 1307803 | 29.92 | 91.02 |
| 20476 | 4722939 | Song 2019 | 447993870 | 2411454 | 29.92 | 91.02 |
| 20476 | 4722940 | Song 2019 | 298868662 | 1651714 | 29.92 | 91.02 |
| 20476 | 4722941 | Song 2019 | 293705649 | 1618066 | 29.92 | 91.02 |
| 20476 | 4722942 | Song 2019 | 372704357 | 2015371 | 29.92 | 91.02 |
| 20476 | 4722943 | Song 2019 | 392040153 | 2126882 | 29.92 | 91.02 |
| 20476 | 4722944 | Song 2019 | 362106828 | 1983255 | 29.92 | 91.02 |
| 20476 | 4722945 | Song 2019 | 337478971 | 1830699 | 29.92 | 91.02 |
| 20476 | 4748643 | Song 2019 | 199827947 | 1115519 | 29.92 | 91.02 |
| 20476 | 4748644 | Song 2019 | 220567715 | 1227536 | 29.92 | 91.02 |
| 20476 | 4748645 | Song 2019 | 199214289 | 1115219 | 29.92 | 91.02 |
| 20476 | 4748646 | Song 2019 | 222397879 | 1230734 | 29.92 | 91.02 |
| 20476 | 4748647 | Song 2019 | 255687994 | 1418234 | 29.92 | 91.02 |
| 20476 | 4748648 | Song 2019 | 223359676 | 1236358 | 29.92 | 91.02 |
| 10541 | 4578926 | Souza 2014 | 1686703041 | 5850205 | -15.61 | -47.74 |
| 10541 | 4578927 | Souza 2014 | 1303666221 | 4898256 | -15.61 | -47.74 |
| 10541 | 4578924 | Souza 2014 | 1221306956 | 5040252 | -15.61 | -47.74 |
| 10541 | 4578925 | Souza 2014 | 1470456607 | 5460208 | -15.61 | -47.74 |
| 10450 | 4577670 | Souza 2016 | 1486805608 | 5420302 | -15.61 | -47.74 |
| 10450 | 4577669 | Souza 2016 | 1562052023 | 5657434 | -15.61 | -47.74 |
| 10450 | 4577671 | Souza 2016 | 1466364965 | 5912295 | -15.61 | -47.74 |
| 10450 | 4577672 | Souza 2016 | 1507973051 | 5708651 | -15.61 | -47.74 |
| 252 | 4523023 | Stackhouse 2015 | 6700261439 | 46206753 | 79.42 | -90.76 |
| 252 | 4523024 | Stackhouse 2015 | 3464483270 | 23875634 | 79.42 | -90.76 |
| 252 | 4523144 | Stackhouse 2015 | 3766167478 | 26022581 | 79.42 | -90.76 |
| 252 | 4523145 | Stackhouse 2015 | 5614599082 | 38480957 | 79.42 | -90.76 |
| 252 | 4521707 | Stackhouse 2015 | 2585421825 | 17894559 | 79.42 | -90.76 |
| 252 | 4521708 | Stackhouse 2015 | 465642025 | 3130077 | 79.42 | -90.76 |
| 252 | 4523775 | Stackhouse 2015 | 4925988000 | 33583779 | 79.42 | -90.76 |
| 252 | 4523146 | Stackhouse 2015 | 2205011588 | 14693575 | 79.42 | -90.76 |
| 252 | 4523776 | Stackhouse 2015 | 812512042 | 5031403 | 79.42 | -90.76 |
| 252 | 4523777 | Stackhouse 2015 | 2406267206 | 15706801 | 79.42 | -90.76 |
| 252 | 4523147 | Stackhouse 2015 | 2541764475 | 16220012 | 79.42 | -90.76 |
| 252 | 4523778 | Stackhouse 2015 | 7078859018 | 48675652 | 79.42 | -90.76 |
| 252 | 4530050 | Stackhouse 2015 | 93390981 | 83249 | 79.42 | -90.76 |
| 252 | 4523148 | Stackhouse 2015 | 3494111865 | 23047923 | 79.42 | -90.76 |
| 252 | 4523779 | Stackhouse 2015 | 4225726829 | 27416020 | 79.42 | -90.76 |
| 252 | 4523025 | Stackhouse 2015 | 2183775720 | 14544508 | 79.42 | -90.76 |
| 223 | 4445996 | Steven 2012 | 116821792 | 312444 | 36.82 | 115.92 |
| 223 | 4445990 | Steven 2012 | 219117356 | 583724 | 36.82 | 115.92 |
| 223 | 4445993 | Steven 2012 | 133555260 | 352417 | 36.82 | 115.92 |
| 223 | 4445994 | Steven 2012 | 254548462 | 683082 | 36.82 | 115.92 |
| 13205 | 4628823 | Su 2017 | 8626384500 | 86263845 | 22.11 | 112.77 |
| 13205 | 4628824 | Su 2017 | 2930463000 | 29304630 | 22.11 | 112.77 |
| 13205 | 4628825 | Su 2017 | 8372459800 | 83724598 | 22.11 | 112.77 |
| 13205 | 4628826 | Su 2017 | 5753935400 | 57539354 | 22.11 | 112.77 |
| 13205 | 4628827 | Su 2017 | 8841671500 | 88416715 | 22.11 | 112.77 |
| 13205 | 4628828 | Su 2017 | 3175243800 | 31752438 | 22.11 | 112.77 |
| 13205 | 4626287 | Su 2017 | 3854190671 | 38541906 | 22.11 | 112.77 |
| 13205 | 4628829 | Su 2017 | 1905620800 | 19056208 | 22.11 | 112.77 |
| 13205 | 4628830 | Su 2017 | 4547269300 | 45472693 | 22.11 | 112.77 |
| 13205 | 4626288 | Su 2017 | 4797390127 | 47973899 | 22.11 | 112.77 |
| 13205 | 4626289 | Su 2017 | 5066531996 | 50665320 | 22.11 | 112.77 |
| 13205 | 4626290 | Su 2017 | 4275596100 | 42755961 | 22.11 | 112.77 |
| 13205 | 4626291 | Su 2017 | 5965667300 | 59656673 | 22.11 | 112.77 |
| 13205 | 4626292 | Su 2017 | 711577500 | 7115775 | 22.11 | 112.77 |
| 13205 | 4628831 | Su 2017 | 10669379700 | 106693797 | 22.11 | 112.77 |
| 13205 | 4626293 | Su 2017 | 2910226200 | 29102262 | 22.11 | 112.77 |
| 13205 | 4626294 | Su 2017 | 3018521300 | 30185213 | 22.11 | 112.77 |
| 13205 | 4626295 | Su 2017 | 4191274000 | 41912740 | 22.11 | 112.77 |
| 375 | 4487653 | Taketani 2015 | 65001425 | 466014 | -6.71 | -38.25 |
| 375 | 4487567 | Taketani 2015 | 52102364 | 371520 | -6.71 | -38.25 |
| 375 | 4487638 | Taketani 2015 | 39262964 | 289162 | -9.22 | -41.09 |
| 375 | 4487345 | Taketani 2015 | 59973310 | 425184 | -9.22 | -41.09 |
| 375 | 4487639 | Taketani 2015 | 71575930 | 503318 | -8.83 | -42.55 |
| 375 | 4487344 | Taketani 2015 | 81657620 | 561526 | -8.83 | -42.55 |
| 14608 | 4654026 | Taketani 2017 | 908651569 | 4902736 | 31.03 | 34.90 |
| 14608 | 4654027 | Taketani 2017 | 523421685 | 2840653 | 31.03 | 34.90 |
| 14608 | 4654028 | Taketani 2017 | 572066482 | 3129422 | 31.03 | 34.90 |
| 14608 | 4654029 | Taketani 2017 | 503468033 | 2725118 | 30.87 | 34.77 |
| 14608 | 4654030 | Taketani 2017 | 531699676 | 2915911 | 30.87 | 34.77 |
| 14608 | 4654031 | Taketani 2017 | 461408862 | 2528610 | 30.87 | 34.77 |
| 14608 | 4654021 | Taketani 2017 | 1811246657 | 9681819 | 33.00 | 35.23 |
| 14608 | 4654022 | Taketani 2017 | 830740317 | 4484452 | 33.00 | 35.23 |
| 14608 | 4654023 | Taketani 2017 | 711633780 | 3762792 | 31.70 | 35.05 |
| 14608 | 4654024 | Taketani 2017 | 977801012 | 5346169 | 31.70 | 35.05 |
| 14608 | 4654025 | Taketani 2017 | 698042789 | 3808872 | 31.70 | 35.05 |
| 14608 | 4654032 | Taketani 2017 | 735731341 | 4053435 | 33.00 | 35.23 |
| 10865 | 4582785 | Tripathi 2016 | 890482723 | 5107828 | 4.96 | 117.82 |
| 10865 | 4582786 | Tripathi 2016 | 964778783 | 5523247 | 4.95 | 117.81 |
| 10865 | 4582787 | Tripathi 2016 | 989118182 | 5657390 | 4.96 | 117.80 |
| 10865 | 4582788 | Tripathi 2016 | 934244819 | 5365741 | 4.97 | 117.79 |
| 10865 | 4582789 | Tripathi 2016 | 1030019164 | 5840128 | 4.97 | 117.82 |
| 10865 | 4582790 | Tripathi 2016 | 855486915 | 4892887 | 4.93 | 117.95 |
| 10865 | 4582791 | Tripathi 2016 | 885006509 | 5039711 | 5.09 | 117.64 |
| 10865 | 4582792 | Tripathi 2016 | 800887970 | 4584476 | 5.10 | 117.63 |
| 10865 | 4582793 | Tripathi 2016 | 958840711 | 5460061 | 5.10 | 117.65 |
| 10865 | 4582794 | Tripathi 2016 | 810908299 | 4608250 | 5.02 | 117.75 |
| 10865 | 4582795 | Tripathi 2016 | 960144683 | 5484909 | 4.99 | 117.86 |
| 10865 | 4582796 | Tripathi 2016 | 1005523825 | 5786291 | 4.93 | 117.98 |
| 10865 | 4582797 | Tripathi 2016 | 703067533 | 4000557 | 5.02 | 117.79 |
| 10865 | 4582798 | Tripathi 2016 | 953239023 | 5415884 | 5.03 | 117.79 |
| 10865 | 4582799 | Tripathi 2016 | 873743523 | 4972424 | 5.09 | 117.68 |
| 10865 | 4582800 | Tripathi 2016 | 876592324 | 5014637 | 5.08 | 117.71 |
| 10865 | 4582801 | Tripathi 2016 | 1251623517 | 7246813 | 5.08 | 117.71 |
| 10865 | 4582802 | Tripathi 2016 | 1102244080 | 6379859 | 5.08 | 117.78 |
| 10865 | 4582803 | Tripathi 2016 | 934349523 | 5355308 | 5.08 | 117.78 |
| 10865 | 4582804 | Tripathi 2016 | 961687707 | 5531464 | 4.49 | 117.77 |
| 10865 | 4582805 | Tripathi 2016 | 883442267 | 5106138 | 4.50 | 117.78 |
| 10865 | 4582806 | Tripathi 2016 | 1251177168 | 7203353 | 4.49 | 117.72 |
| 10865 | 4582807 | Tripathi 2016 | 935187469 | 5409688 | 4.50 | 117.73 |
| 10865 | 4582808 | Tripathi 2016 | 1030529654 | 5822722 | 4.94 | 117.95 |
| 21063 | 4728410 | Tripathi 2019 | 4599271544 | 30116638 | 64.84 | -163.72 |
| 21063 | 4728411 | Tripathi 2019 | 4146316438 | 27156447 | 64.84 | -163.72 |
| 21063 | 4728412 | Tripathi 2019 | 4132713492 | 27236603 | 64.84 | -163.72 |
| 21063 | 4728413 | Tripathi 2019 | 4476642964 | 29113100 | 64.84 | -163.72 |
| 21063 | 4728414 | Tripathi 2019 | 4167049930 | 27237827 | 64.84 | -163.72 |
| 21063 | 4728415 | Tripathi 2019 | 6063956073 | 39426142 | 64.84 | -163.72 |
| 21063 | 4728416 | Tripathi 2019 | 4384345915 | 28530634 | 64.84 | -163.72 |
| 21063 | 4728417 | Tripathi 2019 | 6082753681 | 39745902 | 64.84 | -163.72 |
| 21063 | 4728418 | Tripathi 2019 | 3924772579 | 25686558 | 64.84 | -163.72 |
| 21063 | 4728419 | Tripathi 2019 | 4692718086 | 30663039 | 64.84 | -163.72 |
| 21063 | 4728420 | Tripathi 2019 | 5236566801 | 34294459 | 64.84 | -163.72 |
| 21063 | 4728421 | Tripathi 2019 | 5170572351 | 33813905 | 64.84 | -163.72 |
| 4192 | 4450125 | Tveit 2012 | 41875887 | 103366 | 78.94 | 11.82 |
| 4192 | 4450126 | Tveit 2012 | 41420719 | 101781 | 78.94 | 11.82 |
| 4192 | 4450127 | Tveit 2012 | 56221307 | 136679 | 78.94 | 11.82 |
| 4192 | 4450124 | Tveit 2012 | 36339223 | 89359 | 78.93 | 11.94 |
| 4192 | 4450123 | Tveit 2012 | 43926749 | 108237 | 78.93 | 11.94 |
| 15251 | 4667027 | Van_Goethem 2018 | 2354568638 | 7730414 | -76.87 | 161.75 |
| 15251 | 4667031 | Van_Goethem 2018 | 2891256021 | 9464484 | -76.11 | 162.01 |
| 15251 | 4667022 | Van_Goethem 2018 | 5138962533 | 18261157 | -77.01 | 162.40 |
| 15251 | 4667024 | Van_Goethem 2018 | 2052600655 | 6407139 | -76.78 | 161.45 |
| 15251 | 4667025 | Van_Goethem 2018 | 2435180252 | 8126681 | -76.82 | 162.11 |
| 15251 | 4667018 | Van_Goethem 2018 | 2795914895 | 9469426 | -76.16 | 162.02 |
| 15251 | 4667020 | Van_Goethem 2018 | 2551174732 | 8206577 | -77.02 | 161.78 |
| 15251 | 4667033 | Van_Goethem 2018 | 2681821581 | 8846170 | -77.01 | 161.77 |
| 15251 | 4667023 | Van_Goethem 2018 | 3137282983 | 10765138 | -77.02 | 161.74 |
| 15251 | 4667029 | Van_Goethem 2018 | 3218382203 | 11178757 | -77.02 | 161.71 |
| 15251 | 4667021 | Van_Goethem 2018 | 2747112378 | 8850331 | -77.03 | 161.67 |
| 15251 | 4667028 | Van_Goethem 2018 | 2822157282 | 9246318 | -77.02 | 161.71 |
| 15251 | 4667019 | Van_Goethem 2018 | 3056039766 | 10609771 | -77.02 | 161.76 |
| 15251 | 4667036 | Van_Goethem 2018 | 3301592388 | 10510936 | -76.97 | 161.16 |
| 15251 | 4667030 | Van_Goethem 2018 | 2536587261 | 8204285 | -76.95 | 161.40 |
| 15251 | 4667026 | Van_Goethem 2018 | 2557875769 | 8284819 | -77.05 | 161.36 |
| 15251 | 4667035 | Van_Goethem 2018 | 1213230641 | 4331033 | -77.65 | 163.12 |
| 15251 | 4667034 | Van_Goethem 2018 | 1149752218 | 4023268 | -76.65 | 161.09 |
| 15251 | 4667032 | Van_Goethem 2018 | 1120336883 | 3980751 | -76.73 | 161.01 |
| 11442 | 4600900 | Weigold 2016 | 887511049 | 3172850 | 48.51 | 9.04 |
| 11442 | 4600902 | Weigold 2016 | 285329764 | 988295 | 48.51 | 9.04 |
| 11442 | 4600904 | Weigold 2016 | 735268782 | 2608969 | 48.51 | 9.04 |
| 11442 | 4600906 | Weigold 2016 | 1120730678 | 4354330 | 48.51 | 9.04 |
| 11442 | 4600908 | Weigold 2016 | 1006186248 | 3591383 | 48.51 | 9.04 |
| 11442 | 4600910 | Weigold 2016 | 1155439877 | 4680725 | 48.51 | 9.04 |
| 11296 | 4600198 | Zafra 2016 | 277226894 | 806947 | 19.40 | -98.05 |
| 11296 | 4600199 | Zafra 2016 | 242164452 | 738035 | 19.40 | -98.05 |
| 11296 | 4600200 | Zafra 2016 | 258792943 | 780405 | 19.40 | -98.05 |
| 11296 | 4658539 | Zafra 2016 | 541528045 | 2034720 | 19.40 | -98.05 |
| 11296 | 4658538 | Zafra 2016 | 618259364 | 2202675 | 19.40 | -98.05 |
| 11296 | 4658540 | Zafra 2016 | 589633606 | 2197197 | 19.40 | -98.05 |
| 4184 | 4520354 | Zarraonaindia 2015 | 43380079 | 172829 | 41.02 | -72.53 |
| 4184 | 4520360 | Zarraonaindia 2015 | 52938410 | 210910 | 41.01 | -72.53 |
| 4184 | 4520362 | Zarraonaindia 2015 | 29886068 | 119068 | 41.02 | -72.53 |
| 4184 | 4520368 | Zarraonaindia 2015 | 59765359 | 238109 | 41.02 | -72.53 |
| 4184 | 4520376 | Zarraonaindia 2015 | 64991681 | 258931 | 41.02 | -72.53 |
| 4184 | 4520378 | Zarraonaindia 2015 | 73428544 | 292544 | 41.02 | -72.53 |
| 4184 | 4520380 | Zarraonaindia 2015 | 58978474 | 234974 | 41.01 | -72.53 |
| 4184 | 4520384 | Zarraonaindia 2015 | 69949935 | 278685 | 41.01 | -72.53 |
| 4184 | 4520386 | Zarraonaindia 2015 | 58404688 | 232688 | 41.01 | -72.53 |
| 4184 | 4520390 | Zarraonaindia 2015 | 72401201 | 288451 | 41.01 | -72.53 |
| 4184 | 4520392 | Zarraonaindia 2015 | 71699405 | 285655 | 41.02 | -72.53 |
| 4184 | 4520356 | Zarraonaindia 2015 | 67581750 | 269250 | 41.01 | -72.49 |
| 4184 | 4520358 | Zarraonaindia 2015 | 53897230 | 214730 | 41.01 | -72.49 |
| 4184 | 4520364 | Zarraonaindia 2015 | 57627592 | 229592 | 41.01 | -72.49 |
| 4184 | 4520366 | Zarraonaindia 2015 | 43091931 | 171681 | 41.03 | -72.46 |
| 4184 | 4520372 | Zarraonaindia 2015 | 63085085 | 251335 | 41.03 | -72.46 |
| 4184 | 4520374 | Zarraonaindia 2015 | 105308305 | 419555 | 41.01 | -72.49 |
| 4184 | 4520382 | Zarraonaindia 2015 | 29707607 | 118357 | 41.03 | -72.46 |
| 4184 | 4520388 | Zarraonaindia 2015 | 57175290 | 227790 | 41.01 | -72.49 |
| 4184 | 4520370 | Zarraonaindia 2015 | 82318964 | 327964 | 41.06 | -75.45 |
| 6860 | 4544233 | Žifčáková 2017 | 1641087512 | 4523921 | 49.04 | 13.62 |
| 6860 | 4627252 | Žifčáková 2017 | 4262182344 | 9380309 | 49.04 | 13.62 |

**Soil metagenome reference list:**

1. Albright, M.B. et al. Short-term transcriptional response of microbial communities to nitrogen fertilization in a pine forest soil. Appl. Environ. Microbiol. 84, e00598-18 (2018).

2. Andreote, F.D. et al. The microbiome of Brazilian mangrove sediments as revealed by metagenomics. PloS one 7, e38600 (2012).

3. Argiroff, W.A., Zak, D.R., Lanser, C.M. & Wiley, M.J. Microbial Community Functional Potential and Composition Are Shaped by Hydrologic Connectivity in Riverine Floodplain Soils. Microb Ecol 73, 630-644 (2017).

4. Bach, E.M., Williams, R.J., Hargreaves, S.K., Yang, F. & Hofmockel, K.S. Greatest soil microbial diversity found in micro-habitats. Soil Biology and Biochemistry 118, 217-226 (2018).

5. Bell, T.H. et al. A Diverse Soil Microbiome Degrades More Crude Oil than Specialized Bacterial Assemblages Obtained in Culture. Appl Environ Microbiol 82, 5530-41 (2016).

6. Castaneda, L.E. & Barbosa, O. Metagenomic analysis exploring taxonomic and functional diversity of soil microbial communities in Chilean vineyards and surrounding native forests. PeerJ 5, e3098 (2017).

7. Chang, H.X., Haudenshield, J.S., Bowen, C.R. & Hartman, G.L. Metagenome-Wide Association Study and Machine Learning Prediction of Bulk Soil Microbiome and Crop Productivity. Front Microbiol 8, 519 (2017).

8. Chen, H. et al. One-time nitrogen fertilization shifts switchgrass soil microbiomes within a context of larger spatial and temporal variation. PloS one 14, e0211310 (2019).

9. Choi, S. et al. Effect of experimental soil disturbance and recovery on structure and function of soil community: a metagenomic and metagenetic approach. Sci Rep 7, 2260 (2017).

10. Cline, L.C., Zak, D.R., Upchurch, R.A., Freedman, Z.B. & Peschel, A.R. Soil microbial communities and elk foraging intensity: implications for soil biogeochemical cycling in the sagebrush steppe. Ecol Lett 20, 202-211 (2017).

11. Cline, L.C., Zak, D.R., Upchurch, R.A., Freedman, Z.B. & Peschel, A.R. Soil microbial communities and elk foraging intensity: implications for soil biogeochemical cycling in the sagebrush steppe. Ecology letters 20, 202-211 (2017).

12. Cobo-Diaz, J.F. et al. Metagenomic assessment of the potential microbial nitrogen pathways in the rhizosphere of a mediterranean forest after a wildfire. Microb Ecol 69, 895-904 (2015).

13. Delmont, T.O. et al. Structure, fluctuation and magnitude of a natural grassland soil metagenome. ISME J 6, 1677-87 (2012).

14. Dini-Andreote, F. et al. Dynamics of bacterial community succession in a salt marsh chronosequence: evidences for temporal niche partitioning. ISME J 8, 1989-2001 (2014).

15. Fierer, N. et al. Comparative metagenomic, phylogenetic and physiological analyses of soil microbial communities across nitrogen gradients. ISME J 6, 1007-17 (2012).

16. Fierer, N. et al. Cross-biome metagenomic analyses of soil microbial communities and their functional attributes. Proceedings of the National Academy of Sciences 109, 21390-21395 (2012).

17. Fonseca, J.P. et al. Contrasting the microbiomes from forest rhizosphere and deeper bulk soil from an Amazon rainforest reserve. Gene 642, 389-397 (2018).

18. Fraser, M.W., Gleeson, D., Grierson, P.F., Laverock, B. & Kendrick, G.A. Metagenomic evidence of microbial community responsiveness to phosphorus and salinity gradients in seagrass sediments. Frontiers in microbiology 9, 1703 (2018).

19. Gibbons, S.M. et al. Invasive Plants Rapidly Reshape Soil Properties in a Grassland Ecosystem. mSystems 2 (2017).

20. Guo, J., Cole, J.R., Zhang, Q., Brown, C.T. & Tiedje, J.M. Microbial Community Analysis with Ribosomal Gene Fragments from Shotgun Metagenomes. Appl Environ Microbiol 82, 157-66 (2016).

21. Howe, A., Yang, F., Williams, R.J., Meyer, F. & Hofmockel, K.S. Identification of the Core Set of Carbon-Associated Genes in a Bioenergy Grassland Soil. PLoS One 11, e0166578 (2016).

22. Howe, A.C. et al. Tackling soil diversity with the assembly of large, complex metagenomes. Proceedings of the National Academy of Sciences 111, 4904-4909 (2014).

23. Hultman, J. et al. Multi-omics of permafrost, active layer and thermokarst bog soil microbiomes. Nature 521, 208-12 (2015).

24. Kavamura, V.N., Taketani, R.G., Ferreira, C., de Melo, I.S. & Mendes, R. The role of species turnover in structuring bacterial communities in a local scale in the cactus rhizosphere. Plant and Soil 425, 101-112 (2018).

25. Kerfahi, D. et al. From the High Arctic to the Equator: Do Soil Metagenomes Differ According to Our Expectations? Microb Ecol 77, 168-185 (2019).

26. Kumaresan, D. et al. Microbial Functional Capacity Is Preserved Within Engineered Soil Formulations Used In Mine Site Restoration. Sci Rep 7, 564 (2017).

27. Mendes, L.W., de Lima Brossi, M.J., Kuramae, E.E. & Tsai, S.M. Land-use system shapes soil bacterial communities in Southeastern Amazon region. Applied Soil Ecology 95, 151-160 (2015).

28. Mendes, L.W., Kuramae, E.E., Navarrete, A.A., van Veen, J.A. & Tsai, S.M. Taxonomical and functional microbial community selection in soybean rhizosphere. ISME J 8, 1577-87 (2014).

29. Mendes, L.W., Raaijmakers, J.M., de Hollander, M., Mendes, R. & Tsai, S.M. Influence of resistance breeding in common bean on rhizosphere microbiome composition and function. ISME J 12, 212-224 (2018).

30. Mendes, L.W. et al. Soil-borne microbiome: linking diversity to function. Microb Ecol 70, 255-65 (2015).

31. Meneghine, A.K., Nielsen, S., Varani, A.M., Thomas, T. & Carareto Alves, L.M. Metagenomic analysis of soil and freshwater from zoo agricultural area with organic fertilization. PLoS One 12, e0190178 (2017).

32. Meyer, K.M. et al. Conversion of Amazon rainforest to agriculture alters community traits of methane-cycling organisms. Mol Ecol 26, 1547-1556 (2017).

33. Miura, T. et al. Shifts in the composition and potential functions of soil microbial communities responding to a no-tillage practice and bagasse mulching on a sugarcane plantation. Biology and Fertility of Soils 52, 307-322 (2015).

34. Navarrete, A.A. et al. Multi-Analytical Approach Reveals Potential Microbial Indicators in Soil for Sugarcane Model Systems. PLoS One 10, e0129765 (2015).

35. Navarrete, A.A. et al. Soil microbiome responses to the short-term effects of Amazonian deforestation. Mol Ecol 24, 2433-48 (2015).

36. Nguyen, S.G., Ho, C.T., Lee, J.-H. & Unno, T. Metagenomics analysis of methane metabolisms in manure fertilized paddy soil. The Korean Journal of Microbiology 52, 157-165 (2016).

37. Pan, Y. et al. Impact of long-term N, P, K, and NPK fertilization on the composition and potential functions of the bacterial community in grassland soil. FEMS Microbiol Ecol 90, 195-205 (2014).

38. Pandit, A.S. et al. A snapshot of microbial communities from the Kutch: one of the largest salt deserts in the World. Extremophiles 19, 973-87 (2015).

39. Ridl, J. et al. Plants Rather than Mineral Fertilization Shape Microbial Community Structure and Functional Potential in Legacy Contaminated Soil. Front Microbiol 7, 995 (2016).

40. Smith, C.R. et al. Microbial community responses to soil tillage and crop rotation in a corn/soybean agroecosystem. Ecol Evol 6, 8075-8084 (2016).

41. Song, H.-K. et al. Environmental filtering of bacterial functional diversity along an aridity gradient. Scientific reports 9, 866 (2019).

42. Souza, R.C. et al. Metagenomic analysis reveals microbial functional redundancies and specificities in a soil under different tillage and crop-management regimes. Applied Soil Ecology 86, 106-112 (2015).

43. Souza, R.C. et al. Shifts in taxonomic and functional microbial diversity with agriculture: How fragile is the Brazilian Cerrado? BMC Microbiol 16, 42 (2016).

44. Stackhouse, B.T. et al. Effects of simulated spring thaw of permafrost from mineral cryosol on CO2 emissions and atmospheric CH4 uptake. Journal of Geophysical Research: Biogeosciences 120, 1764-1784 (2015).

45. Steven, B., Gallegos-Graves, L.V., Starkenburg, S.R., Chain, P.S. & Kuske, C.R. Targeted and shotgun metagenomic approaches provide different descriptions of dryland soil microbial communities in a manipulated field study. Environ Microbiol Rep 4, 248-56 (2012).

46. Su, J.-Q. et al. Metagenomic assembly unravel microbial response to redox fluctuation in acid sulfate soil. Soil Biology and Biochemistry 105, 244-252 (2017).

47. Taketani, R.G., Kavamura, V.N., Mendes, R. & Melo, I.S. Functional congruence of rhizosphere microbial communities associated to leguminous tree from Brazilian semiarid region. Environmental Microbiology Reports 7, 95-101 (2015).

48. Tripathi, B.M. et al. The impact of tropical forest logging and oil palm agriculture on the soil microbiome. Mol Ecol 25, 2244-57 (2016).

49. Tripathi, B.M. et al. Distinct taxonomic and functional profiles of microbiome associated with different soil horizons of a moist tussock tundra in Alaska. Frontiers in Microbiology 10, 1442 (2019).

50. Tripathi, B.M. et al. Trends in Taxonomic and Functional Composition of Soil Microbiome Along a Precipitation Gradient in Israel. Microb Ecol 74, 168-176 (2017).

51. Tveit, A., Schwacke, R., Svenning, M.M. & Urich, T. Organic carbon transformations in high-Arctic peat soils: key functions and microorganisms. ISME J 7, 299-311 (2013).

52. Van Goethem, M.W. et al. A reservoir of 'historical' antibiotic resistance genes in remote pristine Antarctic soils. Microbiome 6, 40 (2018).

53. Weigold, P. et al. A metagenomic-based survey of microbial (de)halogenation potential in a German forest soil. Sci Rep 6, 28958 (2016).

54. Zafra, G., Taylor, T.D., Absalon, A.E. & Cortes-Espinosa, D.V. Comparative metagenomic analysis of PAH degradation in soil by a mixed microbial consortium. J Hazard Mater 318, 702-710 (2016).

55. Zarraonaindia, I. et al. The soil microbiome influences grapevine-associated microbiota. MBio 6 (2015).

56. Zifcakova, L. et al. Feed in summer, rest in winter: microbial carbon utilization in forest topsoil. Microbiome 5, 122 (2017).

**Supplementary Table 2. Species loss affecting overall taxonomy and function.** One-factor PERMANOVA main test (pseudo-F statistics) and pairwise test results (pseudo-t statistics) showing the significant difference of microbial taxonomic compositions at genus levels and functional profiles at function levels affected sequential species loss.

| Taxonomy | Source | df | SS | MS | Pseudo-F | P(perm) |
| --- | --- | --- | --- | --- | --- | --- |
|  | Trt | 4 | 2.01E+06 | 5.03E+05 | 824.89 | <0.001 |
|  | Res | 4660 | 2.84E+06 | 609.86 |  |  |
|  | Total | 4664 | 4.85E+06 |  |  |  |
|  | Groups | t | P(perm) |  |  |  |
|  | One, Two^#^ | 31.11 | <0.001 |  |  |  |
|  | One, Five | 39.67 | <0.001 |  |  |  |
|  | One, Eight | 43.28 | <0.001 |  |  |  |
|  | One, All | 46.20 | <0.001 |  |  |  |
|  | Two, Five | 19.40 | <0.001 |  |  |  |
|  | Two, Eight | 23.73 | <0.001 |  |  |  |
|  | Two, All | 27.78 | <0.001 |  |  |  |
|  | Five, Eight | 11.17 | <0.001 |  |  |  |
|  | Five, All | 16.78 | <0.001 |  |  |  |
|  | Eight, All | 11.81 | <0.001 |  |  |  |
| Function | Source | df | SS | MS | Pseudo-F | P(perm) |
|  | Trt | 4 | 4.33E+05 | 1.08E+05 | 99.34 | <0.001 |
|  | Res | 4660 | 5.08E+06 | 1089.80 |  |  |
|  | Total | 4664 | 5.51E+06 |  |  |  |
|  | Groups | t | P(perm) |  |  |  |
|  | One, Two | 10.23 | <0.001 |  |  |  |
|  | One, Five | 12.63 | <0.001 |  |  |  |
|  | One, Eight | 13.42 | <0.001 |  |  |  |
|  | One, All | 14.56 | <0.001 |  |  |  |
|  | Two, Five | 6.91 | <0.001 |  |  |  |
|  | Two, Eight | 8.29 | <0.001 |  |  |  |
|  | Two, All | 10.34 | <0.001 |  |  |  |
|  | Five, Eight | 4.28 | <0.001 |  |  |  |
|  | Five, All | 7.21 | <0.001 |  |  |  |
|  | Eight, All | 6.18 | <0.001 |  |  |  |

^#^denotes the remaining phyla numbers after sequential species loss.

**Supplementary Table 3. Species loss affecting relative abundance of specific functions.** One-factor ANOVA results showing the significant difference of relative abundance of twenty five dominant functional categories at level 1 (mean>0.5%) affected by sequential species loss.

|  | Source | df | SS | MS | F | P |
| --- | --- | --- | --- | --- | --- | --- |
| AAD^#^ | Trt | 4 | 1.92E+02 | 47.94 | 51.01 | <0.001 |
|  | Res | 4660 | 4.38E+03 | 0.94 |  |  |
|  | Total | 4664 | 4.57E+03 |  |  |  |
| CBS | Trt | 4 | 2.38E+02 | 59.59 | 67.39 | <0.001 |
|  | Res | 4660 | 4.12E+03 | 0.88 |  |  |
|  | Total | 4664 | 4.36E+03 |  |  |  |
| CDC | Trt | 4 | 1.86E+01 | 4.65 | 16.32 | <0.001 |
|  | Res | 4660 | 1.33E+03 | 0.29 |  |  |
|  | Total | 4664 | 1.35E+03 |  |  |  |
| CHO | Trt | 4 | 1.70E+03 | 424.23 | 276.59 | <0.001 |
|  | Res | 4660 | 7.15E+03 | 1.53 |  |  |
|  | Total | 4664 | 8.84E+03 |  |  |  |
| CVP | Trt | 4 | 1.49E+02 | 37.21 | 85.34 | <0.001 |
|  | Res | 4660 | 2.03E+03 | 0.44 |  |  |
|  | Total | 4664 | 2.18E+03 |  |  |  |
| CWC | Trt | 4 | 1.37E+02 | 34.16 | 72.95 | <0.001 |
|  | Res | 4660 | 2.18E+03 | 0.47 |  |  |
|  | Total | 4664 | 2.32E+03 |  |  |  |
| DNA | Trt | 4 | 1.02E+02 | 25.41 | 30.89 | <0.001 |
|  | Res | 4660 | 3.83E+03 | 0.82 |  |  |
|  | Total | 4664 | 3.94E+03 |  |  |  |
| FAL | Trt | 4 | 1.74E+00 | 0.44 | 0.99 | 0.410 |
|  | Res | 4660 | 2.05E+03 | 0.44 |  |  |
|  | Total | 4664 | 2.05E+03 |  |  |  |
| IAM | Trt | 4 | 1.44E+01 | 3.59 | 25.64 | <0.001 |
|  | Res | 4660 | 6.53E+02 | 0.14 |  |  |
|  | Total | 4664 | 6.67E+02 |  |  |  |
| MAC | Trt | 4 | 2.34E+02 | 58.47 | 237.58 | <0.001 |
|  | Res | 4660 | 1.15E+03 | 0.25 |  |  |
|  | Total | 4664 | 1.38E+03 |  |  |  |
| MEM | Trt | 4 | 7.95E+01 | 19.88 | 56.53 | <0.001 |
|  | Res | 4660 | 1.64E+03 | 0.35 |  |  |
|  | Total | 4664 | 1.72E+03 |  |  |  |
| MIS | Trt | 4 | 1.97E+01 | 4.91 | 10.91 | <0.001 |
|  | Res | 4660 | 2.10E+03 | 0.45 |  |  |
|  | Total | 4664 | 2.12E+03 |  |  |  |
| MOT | Trt | 4 | 5.90E+01 | 14.74 | 126.19 | <0.001 |
|  | Res | 4660 | 5.44E+02 | 0.12 |  |  |
|  | Total | 4664 | 6.03E+02 |  |  |  |
| NIT | Trt | 4 | 1.47E+01 | 3.66 | 32.40 | <0.001 |
|  | Res | 4660 | 5.27E+02 | 0.11 |  |  |
|  | Total | 4664 | 5.42E+02 |  |  |  |
| NUC | Trt | 4 | 2.82E+01 | 7.05 | 47.64 | <0.001 |
|  | Res | 4660 | 6.89E+02 | 0.15 |  |  |
|  | Total | 4664 | 7.18E+02 |  |  |  |
| PHM | Trt | 4 | 1.86E+01 | 4.66 | 138.04 | <0.001 |
|  | Res | 4660 | 1.57E+02 | 0.03 |  |  |
|  | Total | 4664 | 1.76E+02 |  |  |  |
| POT | Trt | 4 | 1.42E+01 | 3.55 | 60.74 | <0.001 |
|  | Res | 4660 | 2.72E+02 | 0.06 |  |  |
|  | Total | 4664 | 2.86E+02 |  |  |  |
| PPT | Trt | 4 | 1.52E+01 | 3.80 | 4.79 | 0.001 |
|  | Res | 4660 | 3.70E+03 | 0.79 |  |  |
|  | Total | 4664 | 3.71E+03 |  |  |  |
| PRO | Trt | 4 | 4.27E+02 | 106.85 | 19.06 | <0.001 |
|  | Res | 4660 | 2.61E+04 | 5.61 |  |  |
|  | Total | 4664 | 2.66E+04 |  |  |  |
| RCS | Trt | 4 | 5.44E+01 | 13.60 | 63.32 | <0.001 |
|  | Res | 4660 | 1.00E+03 | 0.22 |  |  |
|  | Total | 4664 | 1.05E+03 |  |  |  |
| RES | Trt | 4 | 1.27E+01 | 3.18 | 5.11 | <0.001 |
|  | Res | 4660 | 2.90E+03 | 0.62 |  |  |
|  | Total | 4664 | 2.91E+03 |  |  |  |
| RNA | Trt | 4 | 2.50E+00 | 0.63 | 2.21 | 0.066 |
|  | Res | 4660 | 1.32E+03 | 0.28 |  |  |
|  | Total | 4664 | 1.32E+03 |  |  |  |
| STR | Trt | 4 | 2.09E+02 | 52.33 | 261.58 | <0.001 |
|  | Res | 4660 | 9.32E+02 | 0.20 |  |  |
|  | Total | 4664 | 1.14E+03 |  |  |  |
| SUL | Trt | 4 | 2.81E+01 | 7.04 | 127.05 | <0.001 |
|  | Res | 4660 | 2.58E+02 | 0.06 |  |  |
|  | Total | 4664 | 2.86E+02 |  |  |  |
| VDD | Trt | 4 | 2.90E+02 | 72.60 | 103.46 | <0.001 |
|  | Res | 4660 | 3.27E+03 | 0.70 |  |  |
|  | Total | 4664 | 3.56E+03 |  |  |  |

^#^AAD (Amino Acids and Derivatives), CBS (Clustering-based subsystems), CDC (Cell Division and Cell Cycle), CHO (Carbohydrates), CVP (Cofactors, Vitamins, Prosthetic Groups, Pigments), CWC (Cell Wall and Capsule), DNA (DNA Metabolism), FAL (Fatty Acids, Lipids, and Isoprenoids), IAM (Iron acquisition and metabolism), MAC (Metabolism of Aromatic Compounds), MEM (Membrane Transport), MIS (Miscellaneous), MOT (Motility and Chemotaxis), NIT (Nitrogen Metabolism), NUC (Nucleosides and Nucleotides), PHM (Phosphorus Metabolism), POT (Potassium metabolism), PPT (Phages, Prophages, Transposable elements, Plasmids), PRO (Protein Metabolism), RCS (Regulation and Cell signaling), RES (Respiration), RNA (RNA Metabolism), STR (Stress Response), SUL (Sulfur Metabolism), VDD (Virulence, Disease and Defense).

**Supplementary Table 4. Species loss affecting specific functional beta-diversity.** One-factor PERMANOVA results showing the significant difference of beta-diversity at function levels for twenty five dominant functional categories at level 1 (mean>0.5%) affected by sequential species loss.

|  | Source | df | SS | MS | Pseudo-F | P(perm) |
| --- | --- | --- | --- | --- | --- | --- |
| AAD^#^ | Trt | 4 | 4.14E+05 | 1.03E+05 | 93.00 | <0.001 |
|  | Res | 4660 | 5.18E+06 | 1111.70 |  |  |
|  | Total | 4664 | 5.59E+06 |  |  |  |
| CBS | Trt | 4 | 4.54E+05 | 1.14E+05 | 96.31 | <0.001 |
|  | Res | 4660 | 5.49E+06 | 1178.70 |  |  |
|  | Total | 4664 | 5.95E+06 |  |  |  |
| CDC | Trt | 4 | 8.30E+05 | 2.08E+05 | 121.00 | <0.001 |
|  | Res | 4660 | 7.99E+06 | 1715.60 |  |  |
|  | Total | 4664 | 8.83E+06 |  |  |  |
| CHO | Trt | 4 | 5.93E+05 | 1.48E+05 | 122.56 | <0.001 |
|  | Res | 4660 | 5.63E+06 | 1209.20 |  |  |
|  | Total | 4664 | 6.23E+06 |  |  |  |
| CVP | Trt | 4 | 4.54E+05 | 1.14E+05 | 96.31 | <0.001 |
|  | Res | 4660 | 5.49E+06 | 1178.70 |  |  |
|  | Total | 4664 | 5.95E+06 |  |  |  |
| CWC | Trt | 4 | 4.83E+05 | 1.21E+05 | 97.24 | <0.001 |
|  | Res | 4660 | 5.79E+06 | 1242.60 |  |  |
|  | Total | 4664 | 6.27E+06 |  |  |  |
| DNA | Trt | 4 | 5.11E+05 | 1.28E+05 | 144.09 | <0.001 |
|  | Res | 4660 | 4.13E+06 | 886.86 |  |  |
|  | Total | 4664 | 4.64E+06 |  |  |  |
| FAL | Trt | 4 | 5.04E+05 | 1.26E+05 | 94.59 | <0.001 |
|  | Res | 4660 | 6.21E+06 | 1332.20 |  |  |
|  | Total | 4664 | 6.71E+06 |  |  |  |
| IAM | Trt | 4 | 4.57E+05 | 1.14E+05 | 59.70 | <0.001 |
|  | Res | 4660 | 8.92E+06 | 1913.80 |  |  |
|  | Total | 4664 | 9.38E+06 |  |  |  |
| MAC | Trt | 4 | 4.30E+05 | 1.08E+05 | 84.43 | <0.001 |
|  | Res | 4660 | 5.94E+06 | 1274 |  |  |
|  | Total | 4664 | 6.37E+06 |  |  |  |
| MEM | Trt | 4 | 3.63E+05 | 90801 | 116.45 | <0.001 |
|  | Res | 4660 | 3.63E+06 | 779.73 |  |  |
|  | Total | 4664 | 4.00E+06 |  |  |  |
| MIS | Trt | 4 | 4.50E+05 | 1.12E+05 | 83.21 | <0.001 |
|  | Res | 4660 | 6.30E+06 | 1351.80 |  |  |
|  | Total | 4664 | 6.75E+06 |  |  |  |
| MOT | Trt | 4 | 1.77E+05 | 44272 | 43.63 | <0.001 |
|  | Res | 4660 | 4.73E+06 | 1014.60 |  |  |
|  | Total | 4664 | 4.91E+06 |  |  |  |
| NIT | Trt | 4 | 1.85E+05 | 46295 | 58.66 | <0.001 |
|  | Res | 4660 | 3.68E+06 | 789.16 |  |  |
|  | Total | 4664 | 3.86E+06 |  |  |  |
| NUC | Trt | 4 | 3.35E+05 | 83801 | 99.31 | <0.001 |
|  | Res | 4660 | 3.93E+06 | 843.81 |  |  |
|  | Total | 4664 | 4.27E+06 |  |  |  |
| PHM | Trt | 4 | 3.68E+05 | 92030 | 104.78 | <0.001 |
|  | Res | 4660 | 4.09E+06 | 878.33 |  |  |
|  | Total | 4664 | 4.46E+06 |  |  |  |
| POT | Trt | 4 | 60289 | 15072 | 16.39 | <0.001 |
|  | Res | 4660 | 4.29E+06 | 919.61 |  |  |
|  | Total | 4664 | 4.35E+06 |  |  |  |
| PPT | Trt | 4 | 1.87E+05 | 46657 | 44.81 | <0.001 |
|  | Res | 4660 | 4.85E+06 | 1041.20 |  |  |
|  | Total | 4664 | 5.04E+06 |  |  |  |
| PRO | Trt | 4 | 3.84E+05 | 96024 | 153.53 | <0.001 |
|  | Res | 4660 | 2.91E+06 | 625.42 |  |  |
|  | Total | 4664 | 3.30E+06 |  |  |  |
| RCS | Trt | 4 | 3.32E+05 | 82960 | 54.43 | <0.001 |
|  | Res | 4660 | 7.10E+06 | 1524.10 |  |  |
|  | Total | 4664 | 7.43E+06 |  |  |  |
| RES | Trt | 4 | 2.60E+05 | 64898 | 85.77 | <0.001 |
|  | Res | 4660 | 3.53E+06 | 756.66 |  |  |
|  | Total | 4664 | 3.79E+06 |  |  |  |
| RNA | Trt | 4 | 2.53E+05 | 63189 | 59.64 | <0.001 |
|  | Res | 4660 | 4.94E+06 | 1059.50 |  |  |
|  | Total | 4664 | 5.19E+06 |  |  |  |
| STR | Trt | 4 | 5.02E+05 | 1.26E+05 | 96.94 | <0.001 |
|  | Res | 4660 | 6.03E+06 | 1294.70 |  |  |
|  | Total | 4664 | 6.54E+06 |  |  |  |
| SUL | Trt | 4 | 2.77E+05 | 69238 | 53.78 | <0.001 |
|  | Res | 4660 | 6.00E+06 | 1287.40 |  |  |
|  | Total | 4664 | 6.28E+06 |  |  |  |
| VDD | Trt | 4 | 2.10E+05 | 52580 | 58.24 | <0.001 |
|  | Res | 4660 | 4.21E+06 | 902.86 |  |  |
|  | Total | 4664 | 4.42E+06 |  |  |  |

^#^AAD (Amino Acids and Derivatives), CBS (Clustering-based subsystems), CDC (Cell Division and Cell Cycle), CHO (Carbohydrates), CVP (Cofactors, Vitamins, Prosthetic Groups, Pigments), CWC (Cell Wall and Capsule), DNA (DNA Metabolism), FAL (Fatty Acids, Lipids, and Isoprenoids), IAM (Iron acquisition and metabolism), MAC (Metabolism of Aromatic Compounds), MEM (Membrane Transport), MIS (Miscellaneous), MOT (Motility and Chemotaxis), NIT (Nitrogen Metabolism), NUC (Nucleosides and Nucleotides), PHM (Phosphorus Metabolism), POT (Potassium metabolism), PPT (Phages, Prophages, Transposable elements, Plasmids), PRO (Protein Metabolism), RCS (Regulation and Cell signaling), RES (Respiration), RNA (RNA Metabolism), STR (Stress Response), SUL (Sulfur Metabolism), VDD (Virulence, Disease and Defense).

Supplementary figure legends

**Supplementary Fig. 1.** **Species recution affecting similarity.** Box plots and mean values (black line) of pairwise Bray-curtis similarity of taxonomic compositions at genus levels (Taxonomy) and functional profiles at function levels (Function) affected by sequential species loss.

**Supplementary Fig. 1.**


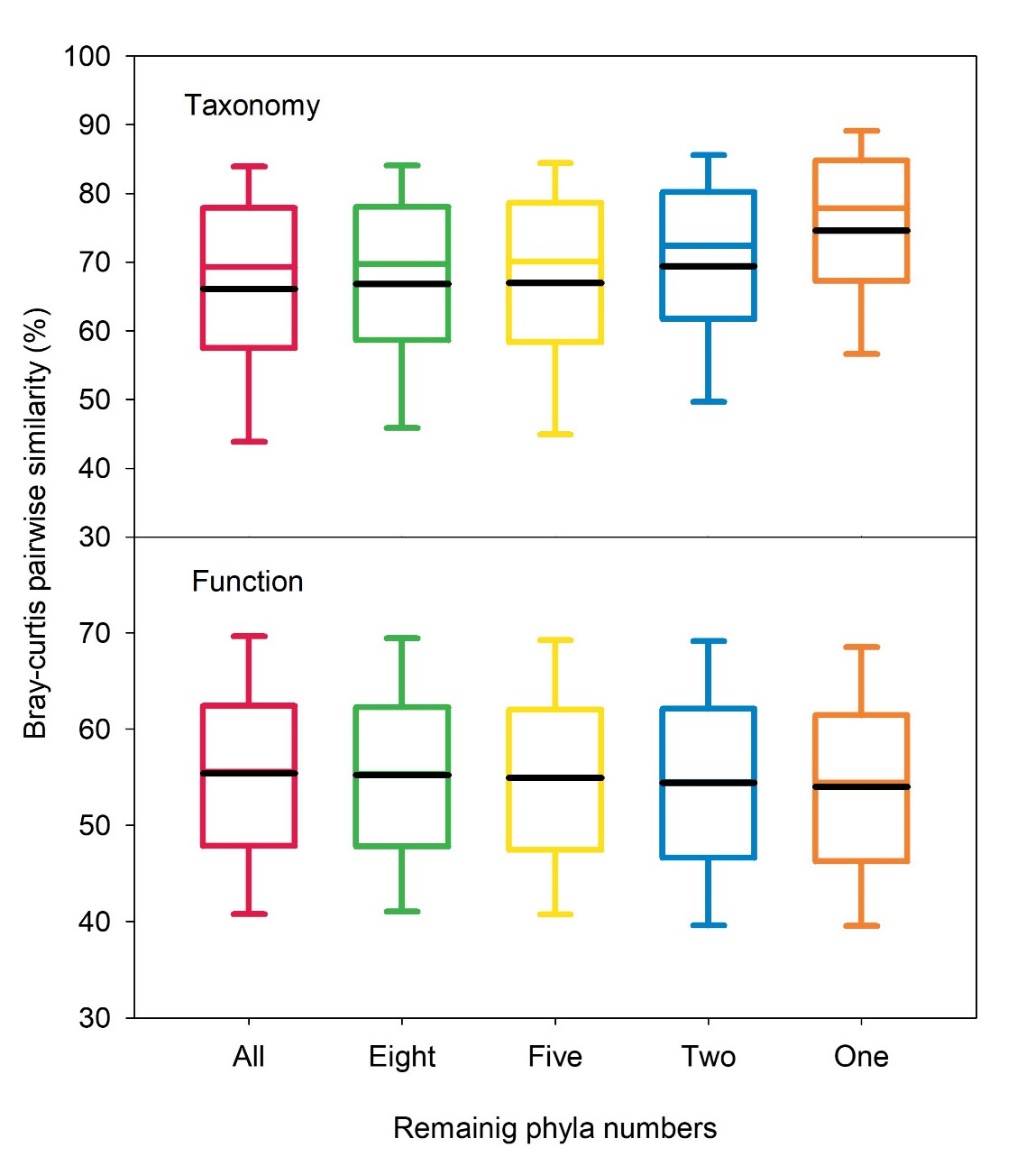

Supplement: Supplementary file 1 [file Data_Sheet_1.DOCX]
